# Quorum sensing in *Aliivibrio wodanis* 06/09/139 and its role in controlling various phenotypic traits

**DOI:** 10.7717/peerj.11980

**Published:** 2021-08-24

**Authors:** Amudha Deepalakshmi Maharajan, Hilde Hansen, Miriam Khider, Nils Peder Willassen

**Affiliations:** 1 Norwegian Structural Biology Center and The Department of Chemistry, Faculty of Science and Technology, UiT-The Arctic University of Norway, Tromsø, Norway; 2 Department of Public Health and Nursing, Faculty of Medicine and Health Sciences, NTNU-Norwegian University of Science and Technology, Trondheim, Norway; 3 Centre for Bioinformatics, Department of Chemistry, Faculty of Science and Technology, UiT-The Arctic University of Norway, Tromsø, Norway

**Keywords:** Quorum sensing, AHL, *Aliivibrio wodanis*, Winter ulcer, Atlantic salmon, Motility, Protease, Cytopathogenicity, Siderophores, CHSE

## Abstract

**Background:**

Quorum Sensing (QS) is a cell-to-cell communication system that bacteria utilize to adapt to the external environment by synthesizing and responding to signalling molecules called autoinducers. The psychrotrophic bacterium *Aliivibrio wodanis* 06/09/139, originally isolated from a winter ulcer of a reared Atlantic salmon, produces the autoinducer N-3-hydroxy-decanoyl-homoserine-lactone (3OHC10-HSL) and encodes the QS systems AinS/R and LuxS/PQ, and the master regulator LitR. However, the role of QS in this bacterium has not been investigated yet.

**Results:**

In the present work we show that 3OHC10-HSL production is cell density and temperature-dependent in *A. wodanis* 06/09/139 with the highest production occurring at a low temperature (6 °C). Gene inactivation demonstrates that AinS is responsible for 3OHC10-HSL production and positively regulated by LitR. Inactivation of *ainS* and *litR* further show that QS is involved in the regulation of growth, motility, hemolysis, protease activity and siderophore production. Of these QS regulated activities, only the protease activity was found to be independent of LitR. Lastly, supernatants harvested from the wild type and the Δ*ainS* and Δ*litR* mutants at high cell densities show that inactivation of QS leads to a decreased cytopathogenic effect (CPE) in a cell culture assay, and strongest attenuation of the CPE was observed with supernatants harvested from the ΔlitR mutant.

**Conclusion:**

*A. wodanis* 06/09/139 use QS to regulate a number of activities that may prove important for host colonization or interactions. The temperature of 6 °C that is in the temperature range at which winter ulcer occurs, plays a role in AHL production and development of CPE on a Chinook Salmon Embryo (CHSE) cell line.

## Introduction

Quorum sensing (QS) is a cell–cell communication mechanism regulated by secretion and accumulation of small diffusible signalling molecules called autoinducers (AI) in a cell density-dependent manner (Bassler, 1999). In Gram-negative bacteria, the most common signalling molecules used for intra-species communication are N-acyl homoserine lactones (AHLs) referred to as AI-1. The AHLs consist of a common homoserine lactone (HSL) ring attached to an acyl side chain with four to 18 carbon atoms and a carbonyl substitution at third carbon (Parsek et al., 1999; Kumari et al., 2006). The type and number of AHLs produced differ between bacteria and also between bacteria of the same species (Purohit et al., 2013; Girard et al., 2019). In addition to the AHLs, bacteria may use autoinducer-2 (AI-2) for both intra- and interspecies QS signalling (Forsyth & Cover, 2000; Schauder et al., 2001; Li et al., 2010; Marques et al., 2011).

QS was first described in *Aliivibrio fischeri*, previously known as *Vibrio fischeri*, and later in other vibrios such as *Vibrio harveyi* (Nealson & Hastings, 1979; Fuqua, Winans & Greenberg, 1994; Freeman, Lilley & Bassler, 2000). *A. fischeri* possesses two AHL based QS systems, the AinS/AinR and LuxI/LuxR, in addition to a LuxS/LuxPQ system where AinS, LuxI and LuxS are the autoinducer synthases and AinR, LuxR and LuxPQ are the receptors. LuxI synthesizes N-3-oxohexanoyl-homoserine lactone (3-oxo-C6-HSL) and AinS synthesizes N-octanoyl- homoserine lactones (C8-HSL) while the LuxS synthesizes a furanosyl borate diester (AI-2) (Lupp & Ruby, 2004; Miyashiro & Ruby, 2012). In *A. fischeri*, the AinS/R and LuxS/LuxPQ QS systems work in parallel to transfer the signal responses to LuxO *via* LuxU. At a low cell density and low autoinducers concentration, the receptors AinR and LuxPQ phosphorylate LuxO. Phosphorylated LuxO activates the transcription of the sRNA *qrr* gene to repress the transcription factor LitR through RNA chaperone Hfq (Lupp et al., 2003). Alternatively, at high cell density, the autoinducers bind to their cognate receptors leading to dephosphorylation of LuxO and *litR* expression. In addition to the phosphorelay system, in *A. fischeri*, LitR directly activates *luxR* expression to regulate bioluminescence production from the *luxICDABEG* operon (Engebrecht & Silverman, 1984; Lupp & Ruby, 2005; Bose, Rosenberg & Stabb, 2008; Verma & Miyashiro, 2013). *A. fischeri* also uses QS to control other activities such as motility, colonization and biofilm formation (Lupp et al., 2003; Lupp & Ruby, 2005; Studer, Mandel & Ruby, 2008; Ray & Visick, 2012).

The marine bacterium *Aliivibrio wodanis* belongs to the genus *Aliivibrio* within the *Vibrionaceae* family (Urbanczyk et al., 2007; Ast, Urbanczyk & Dunlap, 2009). The bacterium is motile and psychrotrophic with the ability to grow at temperatures between 4 and 25 °C in the laboratory (Lunder et al., 1995; Lunder et al., 2000; Soderberg et al., 2019). *A. wodanis* is repeatedly isolated together with *Moritella viscosa* from Atlantic salmons (*Salmo salar*) suffering from winter ulcer disease (Lunder et al., 1995; Benediktsdottir et al., 2000; Lunder et al., 2000; Whitman et al., 2001). The disease has only been reported in reared salmons, and when the sea water temperature drops below 8 °C (Lunder et al., 1995; Benediktsdottir, Helgason & Sigurjonsdottir, 1998; Whitman et al., 2001). The role of *A. wodanis* in pathogenicity of winter ulcer disease is uncertain, and *M. viscosa* is considered to be the main pathogen (Lunder et al., 1995; Benediktsdottir, Helgason & Sigurjonsdottir, 1998; Bruno et al., 1998).

Karlsen et al. (2012) and Karlsen et al. (2014) have attempted to study the interaction between *A. wodanis* and *M. viscosa* using different approaches. The studies suggest that *A. wodanis* may influence the progression of a *M. viscosa* infection. In particular, they show that predisposing Atlantic salmons to *A. wodanis* prior to infection with *M. viscosa* led to lower mortalities (Karlsen et al., 2014). As a follow-up Hjerde et al. (2015) studied the transcriptome of co-cultured *A. wodanis* and *M. viscosa*. In this study the authors concluded that the presence of *A. wodanis* alters the transcriptome of *M. viscosa* and impedes its growth. *A. wodanis* genome encodes bacteriocin, a proteinaceous toxin that inhibits the growth of closely or distantly related bacteria (Hjerde et al., 2015). It was speculated that the expression of bacteriocin in *A. wodanis* might have possibly impeded the growth of *M. viscosa* (Hjerde et al., 2015). Although the contribution of *A. wodanis* in the development of winter ulcer is unclear and may have an alleviating effect on a *M. viscosa* infection, laboratory experiments have shown that Atlantic salmons infected with *A. wodanis* alone is able to cause clinical symptoms such as scale loss, fin rot and internal pathological symptoms such as swollen spleen, peritoneal fat tissues and petecchia in liver (Karlsen et al., 2014). Furthermore, supernatants harvested from high cell density *A. wodanis* cultures induce severe and rapid cytopathogenic effect (CPE) in four differ cell lines of salmonid origins (Karlsen et al., 2014). Hence, the bacterium is able to produce and secrete some yet unknown agent(s) that is cytotoxic to eukaryotic fish cells.

We have previously shown that *A. wodanis* strain 06/09/139 produces N-3-hydroxy-decanoyl-homoserine-lactone (3OHC10-HSL) (Purohit et al., 2013), and encodes the QS systems LuxS/LuxPQ and AinS/AinR and the master regulator LitR (Hjerde et al., 2015). In the study presented here we investigate the QS system of *A. wodanis* by studying the functional roles of the autoinducer synthase AinS and the master regulator LitR. We performed the analyses at different temperatures since *A. wodanis* has been associated with the winter ulcer disease, and also due to the fact that temperature is an important factor for AHL production and QS regulation in the closely related *Aliivibrio salmonicida*, the bacterium known to cause cold-water vibriosis in Atlantic salmons (Bjelland et al., 2012; Hansen et al., 2014; Hansen et al., 2015). Our analysis show that the QS system in *A. wodanis* regulates various phenotypic traits such as motility, growth, hemolysis, protease, siderophore production, as well as cytotoxicity in a cell line. We speculate that QS regulation of various potential virulent factors in *A. wodanis* may play a vital role during winter ulcer development. To our knowledge, this is the first study on the QS system of *A. wodanis*.

## Materials & Methods

### Bacterial strains, plasmids and growth conditions

Bacterial cells and plasmids used in this study are listed in Table 1. The wild type *A. wodanis* 06/09/139 and the constructed *A. wodanis* mutants were grown from glycerol stocks (−80 °C) on Luria-Bertani Agar (Difco BD Diagnostics Sparks, MD, USA) plates containing 1.5% agar (Sigma-Aldrich, St. Louis, MO, USA) and supplemented with a final concentration of 2.5% NaCl (wt/vol) (LA2.5) for 3 days at 12 °C. The Pre-culture of *A. wodanis* was grown overnight in 2 ml of Luria-Bertani broth (LB2.5) at 12 °C and 220 rpm.

**Table 1:** Bacterial strains, plasmids and primers used in this study.

**Strains, plasmids and primers**	**Description**	**Source or reference**
***A. wodanis***		
*A. wodanis* 06/09/139	Wild type from head kidney of Atlantic salmon from west coast of Norway	Karlsen et al. (2014)
Δ*litR*	*A. wodanis*06/09/139 with a complete deletion of *litR* gene	This study
Δ*ainS*	*A. wodanis*06/09/139 with a partial deletion of *ainS* gene	This study
*litR* ^+^	Δ*litR* complemented with the wild type *litR* gene	This study
***E. coli***		
JM109	Competent strain for transformation of pGEM vector with insert	Yanisch-Perron, Vieira & (2005)
SY327	Strain for replicating suicide vector, *λ* pir	Miller & Mekalanos (1988)
S.17-1	Donor strain used for conjugation, *λ* pir	Simon et al. (1983)
**Plasmids**		
pDM4	Suicide vector with *sacB*, cm^R^, R6K origin (*λ*pir)	Milton et al. (1996)
pGEM^®^-T Easy vector	Cloning vector with β-galactosidase, Amp^r^, lacz,3′T overhangs, blue /white screening	Promega
pGEM Δ*litR*	pGEM^®^-T Easy vector with Δ*litR*	This study
pDM4 Δ*litR*	pDM4 with regions flanking the deleted *litR* gene	This study
pDM4 *litR*^+^	pDM4 with flanking regions and full length *litR* gene	This study
pDM4 Δ*ainS*	pDM4 with regions flanking the deleted *ainS* gene	This study
**Primers**		
LitRA-F	ATATACTCGAGTTTACAACAAAAGCGCACCTG	This study
LitRB-R	CATATTTATTTATATCCTTGCCAACAA	This study
LitRC-F	GATATAAATAAATATGTAATATTCAGAACTCAGAAAGTAGATA	This study
LitRD-R	TATAATACTAGTGAGCTTCTTGGTGAAATTGG	This study
LitRG-F	GAGCCACGTAATAAACCAATCATC	This study
LitRH-R	CGTGTTATCGGTGGTGCTATT	This study
AinSA-F	AATAACTCGAGGGCTGATTATACAATAAGGTTGTG	This study
AinSB-R	CTAGATTGTTTAGATCAAATGTTGATA	This study
AinSC-F	GATCTAAACAATCTAGACGAGCCACCAAGATATCAA	This study
AinSD-R	TATATACTAGTCAACCTCCATCCGATCTTTA	This study
AinSG-F	TCACGACGAGAACCAAGACC	This study
AinSH-R	TTAGGTTGATAGCGAGAGCAAAG	This study
NQCAT	TAACGGCAAAAGCACCGCCGGACATCA	Milton et al. (1996)
NQREV	TGTACACCTTAACACTCGCCTATTGTT	Milton et al. (1996)

The *Escherichia coli* (*E. coli*) strains JM109 and S.17-1lambdapir were grown on LA or LB supplemented with 1% (wt/vol) NaCl (LA1 and LB1, respectively) at 37 °C and 220 rpm overnight. The TA plasmid pGEM-T was propagated in *E. coli* JM109 (Promega). The suicide plasmid pDM4 (GenBank: KC795686.1) was propagated in S.17-1lambdapir cells. The *E.coli* JM109 and S.17-1lambdapir transformants were selected on LA1 with 100 µg/ml ampicillin and 25 µg/ml chloramphenicol respectively. The potential transconjugants of *A. wodanis* were selected on LA2.5 supplemented with 2 µg/ml chloramphenicol at 12 °C for 5 days.

A sea water-based medium (SWT) was used in the biofilm and colony morphology assays consists of 5 g/L of Bacto peptone (BD), 3 g/L of yeast extract (Sigma-Aldrich, St. Louis, MO, USA) supplemented with different sea salt (Instant Ocean, Aquarium systems, Sarrebourg, France) concentrations (1.0, 2.8 and 4.0% per litre). For solid SWT plates, 1.5% agar (Sigma-Aldrich, St. Louis, MO, USA) was added. The hemolysin assay was carried on blood agar plates (BA) base no.2 (OXOID, Thermo Scientific) supplemented with 5% blood and 2.5% NaCl. Leibowitch-15 (L-15) medium (Thermo Fisher Scientific, USA) was used for cytotoxicity assay and supplemented with 200 mM L-glutamine, antibiotic-antimycotic solution 100 units/ml penicillin, 100 µg/ml Streptomycin, 250 ng/ml amphotericin B (P/S/A) (Sigma-Aldrich, St. Louis, MO, USA) and fetal bovine serum (FBS) (8%).

### DNA extraction, PCR and sequencing

Genomic DNA was purified using Masterpure™ complete DNA/RNA purification kit (Epicentre, Cambio Ltd., Cambridge) and plasmids were purified using E.Z.N.A^®^ plasmid mini kit (Omega Bio-tek, Inc., Norcross, GA). The DNA concentration was measured using NanoDrop™ 2000c spectrophotometer (Thermo Scientific, DE, USA). The PCR amplification was performed using Phusion^®^ polymerase (Thermo Fisher Scientific, Waltham, MA, USA) or Taq polymerase (Sigma, St. Louis, MO, USA) in a Arktik™ thermal cycler (Thermo Fisher Scientific, USA). Restriction digestion using XhoI and SpeI restriction enzymes and DNA ligation using T4 DNA ligase were performed as recommended by the manufactures and were obtained from New England Biolabs (Ipswich, MA, USA). The PCR products and the digested DNA fragments were separated using agarose gel electrophoresis and extracted using Montage^®^ gel extraction kit (Millipore, MA, USA). DNA sequencing was performed using Big Dye (Applied biosystems, CA, USA). The primers used in the PCR and sequencing reactions were synthesized by Sigma-Aldrich and are listed in Table 1.

### Construction of Δ*litR* and Δ*ainS* mutants

The *litR* (AWOD_I_0419) and *ainS* (AWOD_I_1040) genes were in-frame deleted in *A. wodanis* using allelic exchange as described by others (Milton et al., 1996; Bjelland et al., 2012; Hansen et al., 2015; Khider, Willassen & Hansen, 2018). Briefly, *litR* and *ainS* genes were deleted by amplifying and fusing regions flanking these genes. The upstream (280 bp) and downstream (263 bp) flanking regions of *litR* gene were amplified by primer pairs LitRA/LitRB and LitRC/LitRD respectively. The upstream region contains the start codon (ATG), and the downstream region contains the last three codons (TAA) at the C-terminal end of *litR* gene. The upstream (253 bp) and downstream (271 bp) flanking regions of *ainS* gene were amplified using primer pairs AinSA/AinSB, and AinSC/AinSD, respectively. The upstream PCR product ends before the start codon (ATG), and the downstream PCR product contained the last 149 bp (50 codons) of the *ainS* gene. PCR amplification was performed with an initial denaturation at 98 °C for 30 s (s), followed by 30 cycles of 98 °C for 10 s, 60 °C for 20 s, 72 °C for 30 s, finishing with a final extension at 72 °C for 5 min and storage at 4 °C thereafter. The upstream and downstream PCR products of either *litR* or *ainS* were fused by overlap extension PCR. This overlap PCR was performed by mixing the upstream and downstream PCR products with DNA Phusion polymerase, deoxynucleoside triphosphates (dNTPs) and buffer and cycling for seven times. Then the primer pairs LitRA/D or AinSA/D were added, and 25 more cycles were run (PCR amplification was performed similarly to the stated above). A’overhangs were added to the fused PCR products and ligated into pGEM-T Easy vector. The ligated constructs were transformed into *E. coli* JM109 cells. The inserts (PCR overlap products) were digested from the pGEM plasmid using *SpeI* and *XhoI* restriction enzymes as the primer pairs LitRA/D and AinSA/D contain restriction sites (*SpeI* and *XhoI*) to enable further ligation. The digested fused PCR products were further ligated into corresponding restriction sites of the suicide vector pDM4. The pDM4 plasmid with either *litR* or *ainS* fused PCR product was transformed to *E.coli* S.17-1lambdapir cells. The resulting plasmids were designated as pDM4Δ*litR* and pDM4Δ*ainS*.

The pDM4Δ*litR* and pDM4Δ*ainS* constructs were transferred into wild type *A. wodanis* by bacterial conjugation as described previously (Milton et al., 1996; Bjelland et al., 2012; Khider, Willassen & Hansen, 2018). Briefly, the donor cells *E.coli* S.17-1lambdapir harboring the pDM4Δ*litR* or pDM4Δ*ainS* were grown until mid-exponential phase to OD_600_ (optical density at 600 nm) of 1.0 and the recipient strain (*A. wodanis*) to an early-exponential phase OD_600_ of 2.0. One ml from each culture was centrifuged separately at room temperature and the pellets were separately washed twice with LB1 medium. The washed bacterial pellets were mixed and resuspended in 10 µl of LB1. The resuspended pellet was spotted onto LA1 plates and incubated at 19 °C for 6 h. The plates were further incubated at 12 °C for 48 h. The spotted cells were then resuspended in 2 ml LB2.5 and incubated overnight at 12 °C before plating (20, 40, 60, 80, and 100 µl) on LA2.5 plates with 2 µg/ml chloramphenicol. Potential transconjugants were selected after 3 to 5 days and confirmed using colony PCR. To complete the allelic exchange needed to generate the complete deletion mutants (Δ*ainS* and Δ*litR*) potential transconjugants (*A.wodanis*-pDM4-Δ*ainS* or *A.wodanis*-pDM4-Δ*litR*) were plated on LA2.5 plates with 5% sucrose to allow the second cross over. The cells that were able to grow were selected based on their sensitivity to chloramphenicol. The antibiotics sensitive cells were confirmed by colony PCR and further verified by sequencing.

### Construction of Δ*litR* complementary strain

The complementary strain (*litR*^+^) of the Δ*litR* mutant was constructed by amplifying the full-length wild type *litR* gene using primers LitRA and LitRD. Briefly, the length of both the parental *litR* gene and its flanking region used to generate *litR*^+^ was 1,145 bp, which was amplified from wild type using primers LitRA and LitRD. The PCR product was then cloned into pDM4 using restriction digestion and ligation as described above. The resulting plasmid pDM4*litR*^+^ was transformed into *E.coli* S.17-1lambdapir cells and further transferred to the *A. wodanis* Δ*litR* mutant by bacterial conjugation (described above). The selection and verification of the potential complementary strain were performed as described above. The region that flanks the complemented region (*litR*^+^) after allelic exchange was confirmed using primers LitRG and LitRH. The length of products amplified using LitRG and LitRH was 1,365 bp.

### Preparation of bacterial supernatants for AHL measurements

The wild type *A. wodanis*, Δ*litR* and *litR*^+^ were cultivated in parallel at 6 and 12 °C. The cultures were diluted to a start OD_600_ of 0.001 in a total volume of 60 ml LB2.5 in a 250 ml baffled flasks. The cultures were grown further at the selected temperatures and 220 rpm. Cultures of 1 ml (wild type, Δ*litR* and *litR*^+^) were collected at seven different cell densities in total, six at the log phase (OD_600_ of 0.5, 1.0, 2.0, 3.0, 4.0, 5.0) and one at the stationary phase (8.0). For Δ*ainS* AHL measurements, samples were only harvested at the early stationary phase (OD_600_ of 6.0). The cultures were centrifuged at 13,000× g for 2 min at 4 °C (Heraeus fresco 21; Thermo Scientific, Waltham, MA, USA). Seventy-five microliters of each supernatant were acidified with 4 µl of 1M HCl and stored in three technical replicates at −20 °C before measuring the AHLs. A commercial 3-OH-C10-HSL was used as a standard (Sigma-Aldrich, St. Louis, MO, USA). The sample preparations for High-Performance Liquid Chromatography-Tandem Mass Spectrometry (HPLC-MS/MS) were done as described by others (Purohit et al., 2013; Hansen et al., 2015). Briefly, the acidified supernatants were mixed with three volumes of ethyl acetate (225 µl) and vortexed. The ethyl acetate phase of the three technical replicates was pooled together into a 1 ml 96 well plate and dried in a rotary vacuum centrifuge at −90 °C for 2 h (SpeedVac Savant™ concentrator; Thermo Scientific). The dried samples were dissolved in 150 µl of 20% acetonitrile containing 0.1% formic acid and 660 ng/ml of internal standard 3-oxo-C12-HSL (Sigma-Aldrich, St. Louis, MO, USA).

### HPLC-MS/MS analysis

The HPLC-MS/MS analysis was performed as described in Purohit et al. (2013) and Hansen et al. (2015). Briefly HPLC-MS/MS was performed using an Ascentis Express C18 reversed-phase column (50 × 2.1 mm, 2.7 µm particle size; Sigma). A sample of 20 µl was injected into the column and eluted using 0.1% formic acid in water and 0.1% formic acid in acetonitrile at a flow rate of 200 µl/min. The elution profile obtained was 5% acetonitrile in 30 s, 90% in 300 s and 5% in the next 60 s. The separated compounds were detected by Linear Ion Trap Quadrapole (LTQ) part of the LTQ-Orbitrap (Thermo Fisher Scientific). The LTQ was used in selected reaction monitoring (SRM) mode, and the SRM was divided into two segments. Segment 1 scanned 3OHC10-HSL and segment 2 scanned the internal standard 3O-C12-HSL with a retention time of 0–3.15 min and 3.15–6.00 min, respectively. The ion trap parameters chosen for MS/MS were maximum injection time 50 ms, isowidth 1.0 m/z, collision energy 35, act Q 0.25 and act time 30 ms. The measured AHLs are presented in ng/ml/OD_600_. The AHL measurements at different temperatures were performed twice.

### Motility assay

Motility assay was performed in LA2.5 soft agar plates with 0.25% agar (Bjelland et al., 2012; Khider, Willassen & Hansen, 2018). Pre-cultures of *A. wodanis* wild type, Δ*ainS,* Δ*litR* and *litR*^+^ were diluted 1:100 in LB2.5 and grown overnight at 12 °C to an OD_600_ corresponding to 1.0. Then 2 µl of each culture was spotted onto the LA2.5 soft agar plates and incubated at 6 °C and 12 °C. The diameter of motility zones were measured in millimeters every 24 h for 5 days.

### Siderophore, hemolysis, chitinase and protease assays

Siderophore production was screened using Chromeazurol S (CAS) agar plates as described by others (Lauzon et al., 2008), with the exception that 2.5% of NaCl was used in this study. Culture supernatants harvested at OD_600_ 6.0 (100 µl) from strains grown at 6 and 12 °C were added to six mm wells casted in CAS agar. The CAS agar plates were incubated at 20 °C for 2 days. Hemolysin production was estimated by spotting 2 µl cultures of each strain on BA plates. The protease production was estimated by spotting 2 µl cultures of each strain on LA2.5 agar plates supplemented with 2% skim milk (Sigma-Aldrich, St. Louis, MO, USA). The chitinase assay was performed by spotting 2 µl cultures of each strain on LA2.5 supplemented with 2% colloidal chitin (Sigma-Aldrich, St. Louis, MO, USA) and the plates were stained with 0.5% congo red (Sigma-Aldrich, St. Louis, MO, USA) for 30 min before destaining with 1 M NaCl for 20 min for chitinase zones measurement. All the assays were performed with at least three biological replicates of strains *A. wodanis* wild type, Δ*ainS*, Δ*litR* and *litR*^+^ and were incubated at 6 and 12 °C. The clear zones ratio values for hemolysis, protease and chitinase assays were calculated as clear zone diameter/colony diameter.

### Biofilm and colony morphology assays

The biofilm and colony morphology assays were performed as described previously (Hansen et al., 2014) using SWT media and plates, respectively. Pre-cultures of *A. wodanis* wild type, Δ*ainS*, and Δ*litR* were diluted 1:100 in LB2.5 and grown overnight at 12 °C to an OD_600_ of 1.0. For the biofilm assay, the cultures were further diluted 1:10 in SWT media, and a total volume of 300 µl was added to Falcon 24 well plates (BD Biosciences) and incubated statically at 6 °C. The plates were monitored every 24 h. For the colony morphology assay, a 250 µl of each bacterial culture was harvested by centrifugation, and the pellet was resuspended in 250 µl SWT. Then, 2 µl of each culture was spotted onto SWT plates and incubated at 6 °C for up to 4 weeks. The biofilm formation and colony morphology was visualized using Ziess Primovert microscope at 10x and 4x magnification, respectively and were photographed with AxioCam ERc5s.

### Cytotoxicity assay and crystal violet staining

Chinook salmon embryo (CHSE-214) cells were purchased from American Type Culture Collection (Nicholson & Byrne, 1973). Chinook salmon embryonic (CHSE) cells (passage 55) were grown in L-15. The CHSE cells were seeded 1 × 10^5^ cells/ml in a flat-bottom tissue culture 24-well plates (Falcon; BD Biosciences) and incubated for 48 h at 20 °C. Supernatants of *A. wodanis*, Δ*ainS*, Δ*litR* and *litR*^+^ grown at 6 °C and 12 °C were harvested at OD_600_ of 6.0, 7.0 and 8.0 and filter sterilized through 0.22 µm filter. The 100% confluent fish cells were washed with L-15 without supplements and treated with bacterial supernatants (1:10 to L-15 with antibiotics) before incubating at 12 °C. LB2.5 was used as a negative control. The plates were monitored after 4 and 24 h. The treated CHSE cells were quantified using crystal violet staining. Briefly, the wells with treated CHSE cells were stained with 500 µL of 0.1% crystal violet for 20 min before washing with water. The plates were air-dried for 1 or 2 days, and the cells were dissolved in 96% ethanol before measuring the absorbance at 590 nm (100 µL) using a spectrophotometer (Spectromax, Molecular devices).

All assays were carried out in biological triplicates, unless otherwise indicated. The assays were also performed in two to three independent experiments to validate the results.

## Results

In order to study the roles of QS in *A. wodanis* 06/09/139, we deleted parts of the *ainS* gene (347 of 396 codons) and the *litR* gene (200 of 201 codons) using allelic exchange. A complementary Δ*litR* strain (*litR*^+^) was constructed by re-inserting the full-length copy of the wild type *litR* gene into the Δ*litR* mutant to ensure that observed phenotypes are due to the targeted gene inactivation and not to other factors. Despite several trials, we were unable to rescue the Δ*ainS* mutant. The schematic presentation of the *litR* and *ainS* genes in the genome of *A. wodanis* is shown in Fig. S1.

### The AinS autoinducer synthase is responsible for the production of 3OHC10-HSL

**Figure 1:**
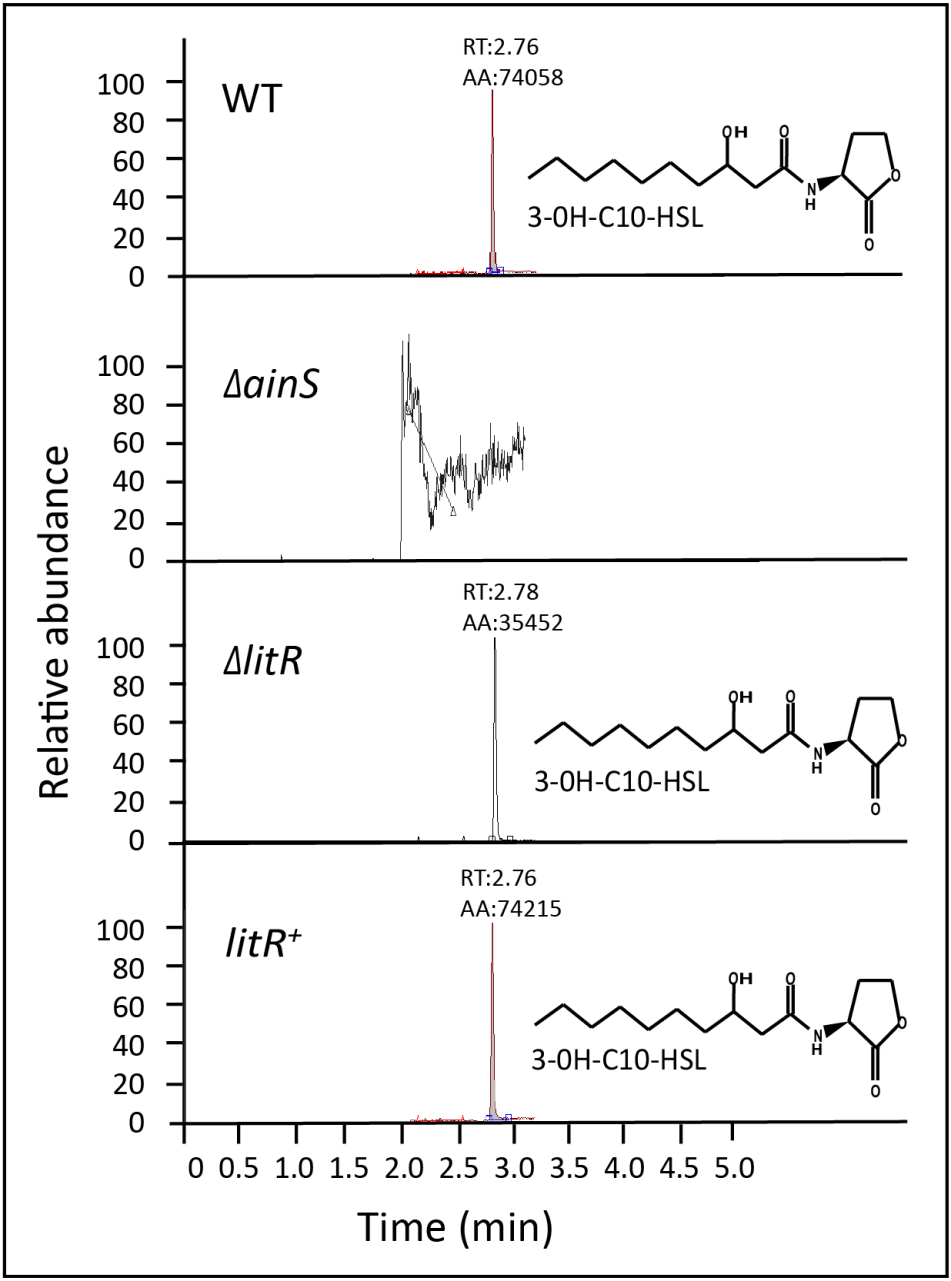
3OHC10-HSL screening in wild type, Δ*ainS*, Δ*litR* and *litR*^+^. HPLC-MS/MS peaks showing the relative abundance of 3OHC10-HSL in supernatants harvested at OD_600_ of 6.0 after growth of the different bacterial strains at 12 °C. LB2.5 was used as a blank. RT: Retention Time, AA: Peak area count.

In previous work, we mapped AHL profiles among members of the *Vibrionaceae* family. Only one single AHL, the 3OHC10-HSL, was identified in *A. wodanis* 06/09/139 (Purohit et al., 2013). In *A. salmonicida*, the autoinducer synthase AinS was responsible for 3OHC10-HSL synthesis (Hansen et al., 2015). Thus our first aim was to verify if 3OHC10-HSL is produced by AinS. To this end, supernatants harvested from the wild type and mutants (Δ*ainS,* Δ*litR* and *litR*^+^) were analyzed by HPLC-MS/MS as previously described (Purohit et al., 2013; Hansen et al., 2015). A peak corresponding to 3OHC10-HSL was present only in supernatants harvested from the wild type, Δ*litR* mutant and the *litR*^+^ (Fig. 1). This suggests that AinS is the autoinducer synthase responsible for 3OHC10-HSL production in *A. wodanis* 06/09/139. Unfortunately, we were not able to complement the *ainS* mutant which could have given absolute proof for AinS being the 3OHC10-HSL synthase in *A. wodanis*. Since *ainS* is the only AHL-linked gene annotated in *A. wodanis* we find other explanations unlikely.

### LitR represses growth of *A. wodanis* at 20 °C

**Figure 2:**
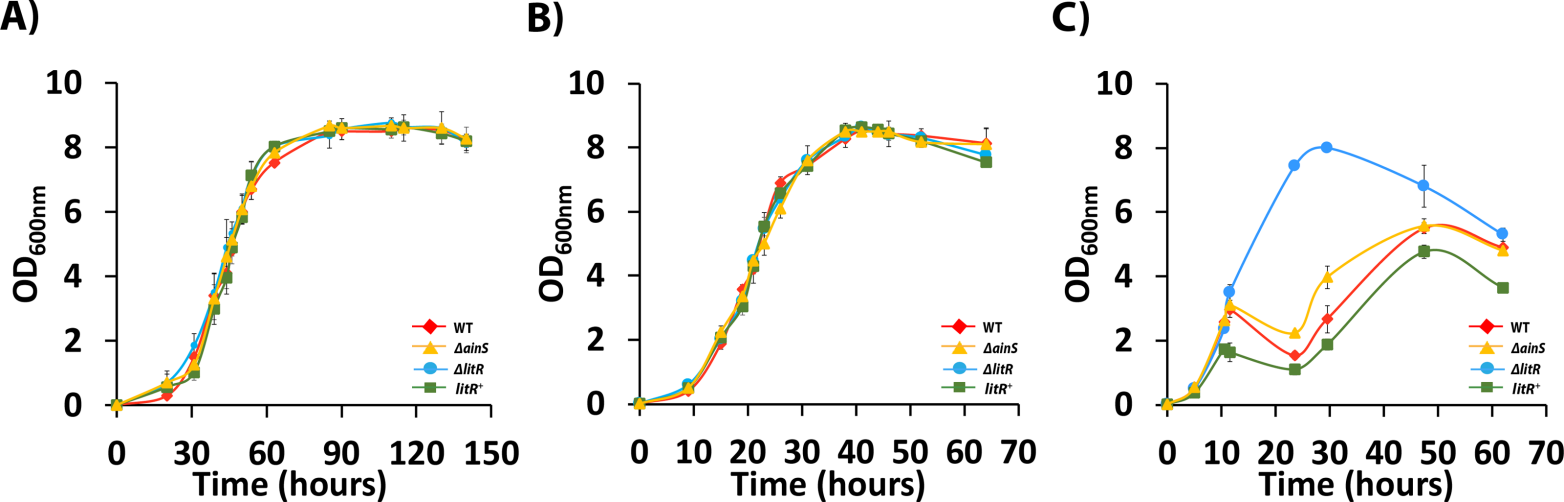
Growth curves of wild type *A. wodanis* 06/09/139, and the isogenic mutants Δ*ainS*, Δ*litR* and *litR*^+^. The strains were grown in LB2.5, 220 rpm at 6 °C (A), 12°C (B) and 20 °C (C). The error bars indicate the standard deviation of three biological replicates.

Mutations are known to affect the growth of bacteria. *A. wodanis* strains grow in a range of 4–25 °C and in a recent study in our lab, the optimal growth temperatures were found to be 12–18 °C (Lunder et al., 1995; Lunder et al., 2000; Soderberg et al., 2019). We, therefore tested the strains ability to grow at three different temperatures (6 °C, 12 °C and 20  °C) within the reported temperature range for *A. wodanis*. As shown in Fig. 2, the *litR* or *ainS* mutations did not alter the growth rate of *A. wodanis* at 6 °C and 12 °C, and all strains reached a maximum OD_600_ of ∼8.0. The strains grew considerably faster at 12 °C than at 6  °C, and the duration of the log/exponential phase (OD_600_ 0.5 to 8.0) for the wild type lasted for 22 h when grown at 12 °C compared to 45 h at 6 °C (Fig. 2). At 20  °C, which is a non-optimal temperature for growth of *A. wodanis* in the laboratory (Soderberg et al., 2019), the wild type and Δ*ainS* mutant showed a growth deficiency, and the growth halted after reaching an OD_600_  of 2.0–3.0 before it finally reached maximum OD_600_ of 5.0–6.0. On the other hand, the Δ*litR* mutant grew steadily and was able to reach an OD_600_ of 8.0. Neither of the strains grew in liquid media (LB2.5) at 25  °C, and after streaking single colonies of the different strains onto blood agar plates (BA2.5) only the Δ*litR* mutant was able to form small colonies when incubated at 25 °C (Fig. S2). Thus, LitR represses the ability of *A. wodanis* to grow at 20 °C and 25 °C.

### 3OHC10-HSL production in *A. wodanis* is cell density and temperature-dependent, and weakly regulated by LitR

**Figure 3:**
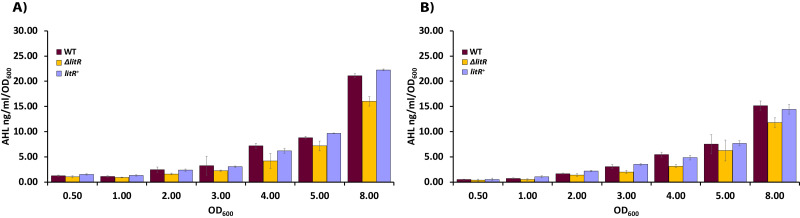
AHL profiling of supernatants harvested from wild type *A. wodanis* 06/09/139, Δ*litR* and *litR*^+^. (A) The 3OHC10-HSL concentrations (ng/ml/OD_600_) were measured in acidified supernatants by HPLC-MS/MS after growth of the different strains at 6 °C and (B) at 12 °C. The error bars indicate the standard deviation of three biological replicates.

To explore the role of temperature on the production of 3OHC10-HSL in *A. wodanis*, we analyzed supernatants harvested from the wild type 06/09/139 at different cell densities after growth at 6 and 12 °C. The HPLC-MS/MS analyses showed that the 3OHC10-HSL production was detectable from the measurements started at OD_600_ of 0.5 and increased along the growth curve in a cell density-dependent manner. The bacterium produced higher concentrations of 3OHC10-HSL when it was grown at 6 °C compared to at 12 °C (*P* < 0.05, by Students *t* test). Highest 3OHC10-HSL concentrations were measured in the stationary phase (OD_600_ of 8.0) where the wild type reached concentrations of 21.06 ± 0.43 ng/ml/OD_600_ and 15.12 ± 0.94 ng/ml/OD_600_ after growth at 6  °C and 12 °C, respectively (Table S1, Fig. 3).

We also analyzed supernatants harvested from the Δ*litR* mutant to examine if LitR is a regulator of AHL production in *A. wodanis,* similar to what has been shown for other aliivibrios (Lupp & Ruby, 2004; Hansen et al., 2015). As shown in Fig. 3, the Δ*litR* mutant produced lower concentrations of 3OHC10-HSL than the wild type did in the stationary phase, and the deletion of *litR* led to an 24% reduction when the maximum concentrations (measured at OD_600_ = 8.0) of the wild type and Δ*litR* were compared at 6 °C (WT = 21.06 ± 0.43 ng/ml/OD_600_ and Δ*litR* = 16.01 ± 0.96 ng/ml/OD_600_; *P* < 0.05 by Students *t* test) and 22% reduction after growth at 12 °C (WT = 15.12 ± 0.94 ng/ml/OD_600_ and Δ*litR* = 11.78 ± 0.94 ng/ml/OD_600_; *P* < 0.05 by Students *t* test). The complementary mutant *litR*^+^ behaved as the wild type with regard to 3OHC10-HSL production.

### Phenotypic traits regulated by QS in *A. wodanis*06/09/139

QS is known to regulate several activities or phenotypic traits in vibrios and allivibrios (Croxatto et al., 2002; Zhu et al., 2002; Lee et al., 2004; Tsou & Zhu, 2010; Bjelland et al., 2012; Khider et al., 2019). Therefore, we analyzed the wild type *A. wodanis* 06/09/139 and QS mutants (Δ*ainS* and Δ*litR*) with regard to motility, protease and siderophore production, hemolysis, chitinase activity, biofilm formation and colony morphology. The experiments were performed at 6  °C and 12 °C to determine if the temperature has an influence on the phenotypic traits exhibited by the wild type, Δ*ainS* and Δ*litR* mutants.

#### Motility

**Figure 4:**
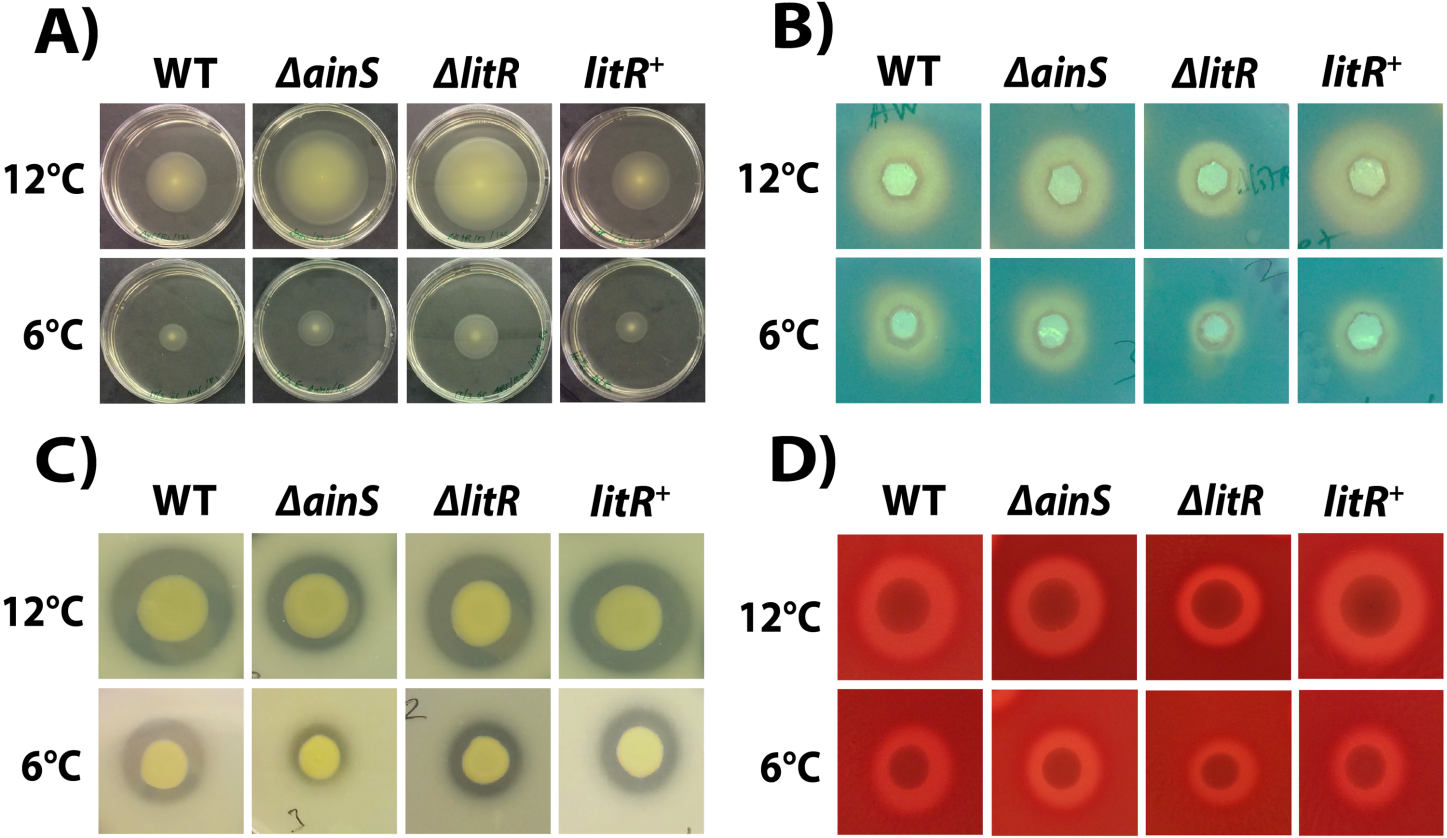
Motility, siderophore- and protease production, and hemolytic activity in *A. wodanis*, Δ*ainS,*Δ*litR* and *litR*^+^ mutants at 6 °C and 12 °C. (A) Soft agar plates showing the motility zones after 2 days. (B) Siderophores produced at OD_600_ of 6.0 visible as yellow halos on CAS agar. (C) Protease production visible as cleared zones on skim milk agar plates. (D) Hemolytic zones on blood agar.

The motility assay showed that the wild type *A. wodanis* 06/09/139 was motile at both 6 °C and 12  °C. The motility of wild type *A. wodanis* was 57% higher at 12 °C compared to at 6  °C (12 °C =42.17 ± 3.19 mm and 6 °C =18.00 ± 0.89 mm; *P* < 0.05 by Students *t* test) (Fig. 4A). The Δ*ainS* and Δ*litR* mutants showed significantly higher motility than the wild type at both temperatures. Compared to wild type, the Δ*litR* mutant showed 27% larger motility zones both at 6 °C (WT = 18.00 ± 0.89 mm and Δ*litR* = 24.58 ± 1.74 mm; *P* < 0.05 by Students *t* test) and at 12 °C (WT = 42.17 ± 3.19 mm and Δ*litR* = 57.67 ± 1.97 mm; *P* < 0.05 by Students *t* test). Similarly, the Δ*ainS* mutant showed 17% larger motility zones at 6  °C (WT = 18.00 ± 0.89 mm and Δ*ainS* = 21.67 ± 1.51 mm; *P* < 0.05 by Students *t* test) and 26% larger zones at 12  °C (WT = 42.17 ± 3.19 mm and Δ*ainS* = 57.17 ± 3.87 mm; *P* < 0.05 by Students *t* test) (Fig. 4A, Table S2).

#### Siderophore production

Siderophores are produced by the bacterium and secreted into the growth medium (Sandy & Butler, 2009). Hence, the activity was analyzed in supernatants harvested at OD_600_ of 6.0 from the wild type and mutants after growth at 6 and 12 °C. The CAS assay showed that supernatants harvested from wild type at 12  °C produced 19% larger zones than supernatants harvested at 6 °C (Fig. 4B, Table S2). Siderophore production was negatively affected by the *litR* mutation, and the zones formed by Δ*litR* mutant supernatants were 19% smaller at 6  °C (WT = 14.00 ± 1.00 mm and Δ*litR* = 11.33 ± 1.53 mm; *P* < 0.05 by Students *t* test) and 29% smaller at 12 °C (WT = 17.33 ± 1.15 mm and Δ*litR* = 12.33 ± 1.15 mm; *P* < 0.05 by Students *t* test) when compared to the zones produced by wild type supernatants. The size of siderophore zones formed by the supernatants from Δ*ainS* mutant was not significantly different from the wild type at neither 6  °C nor at 12 °C (Fig. 4B, Table S2).

#### Protease activity

The protease assay showed that the wild type *A. wodanis* 06/09/139 was able to cleave the skim milk embedded in the agar (Fig. 4C). When compared to wild type, the average proteolytic zone ratio of the Δ*ainS* mutant was 16% smaller at 6 °C (WT = 1.47 ± 0.20 and Δ*ainS* = 1.23 ± 0.08; *P* < 0.05 by Students *t* test) and 17% smaller at 12 °C (WT = 1.78 ± 0.15 and Δ*ainS* = 1.47 ± 0.07; *P* <0.05 by Students *t* test). The proteolytic zones produced by the Δ*litR* mutant were not significantly different from the ones produced by the wild type at neither 6 °C nor 12 °C (Fig. 4C, Table S2).

#### Hemolytic activity

The hemolysis assay showed that the wild type *A. wodanis* 06/09/139 was hemolytic (Fig. 4D). The Δ*litR* mutant produced hemolytic zones ratio that were 10% smaller than the corresponding zones produced by the wild type at 6 °C (WT = 1.76 ± 0.07 and Δ*litR* = 1.58 ± 0.07; *P* < 0.05 Students *t* test) and 10% smaller at 12 °C (WT = 1.84 ± 0.03 and Δ*litR* = 1.66 ± 0.06; *P* < 0.05 by Students *t* test). Deletion of *ainS* had no significant effects on the hemolytic activity, as both Δ*ainS* and wild type produced similar hemolytic zones on the blood agar plates (*P* > 0.05 by Students *t* test) (Fig. 4D, Table S2).

#### Chitinase activity

The assay showed no significant differences (*P* > 0.05 by Students *t* test) in chitinase zones between the wild type and mutants (Δ*ainS* and Δ*litR*) (Fig. S3, Table S2).

#### Colony morphology and biofilm formation

The ability of the mutants to form biofilm and colony morphology was analyzed in the SWT media with different salt conditions. The strains (*A. wodanis*, ΔainS and ΔlitR) did not form biofilm (Fig. S4). Similarly, on the SWT plates, the colonies of Δ*ainS* and Δ*litR* looked smooth similar to the wild-type with no rugosity both microscopically and macroscopically (Fig. S5).

### Cytopathogenic effect on Chinook salmon embryonic cells (CHSE)

**Figure 5:**
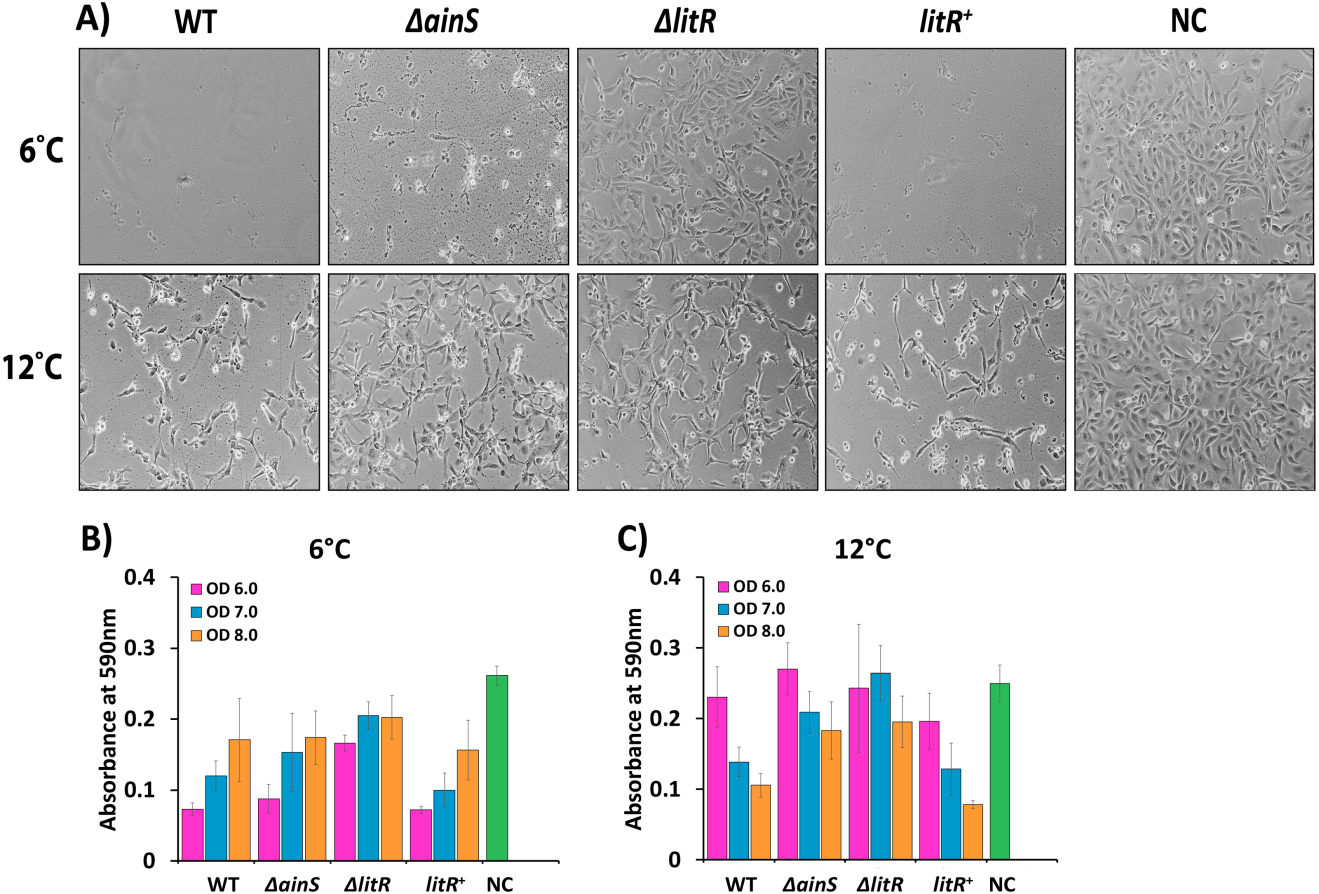
Cytopathogenic effect (CPE) in CHSE cells inoculated with supernatants of wild type *A. wodanis*, Δ*ainS*, Δ*litR* mutants and *litR*^+^ harvested at 6 and 12 °C. (A) CPE observed in CHSE cells treated with supernatants harvested at OD_600_ of 6.0 from strains grown at 6 °C (top) and 12 °C (bottom). The images were taken after 24 h incubation at 12 °C with a Nikon Eclipse TS100 Inverted Phase contrast Microscope at 10x magnification. The bar charts represent the absorbance measured after crystal violet staining the remaining attached CHSE cells after being exposed to supernatants harvested at OD_600_ of 6.0–8.0 from strains grown at 6 °C (B) and 12 °C (C). NC denotes negative control. The error bars indicate the standard deviation of three biological replicates.

To test whether QS affects the cytopathogenic effect (CPE) of *A. wodanis* 06/09/139, CHSE cells were treated with supernatants harvested at different cell densities (OD_600_ of 6.0, 7.0 and 8.0) from the wild type and mutants (Δ*ainS* and Δ*litR*) after growth at 6 and 12 °C. The CPE was observed microscopically (Fig. 5A), and cells that survived and remained attached to the substratum were thereafter quantified using a crystal violet staining method (Figs. 5B and 5C). Supernatants harvested from the wild type had a CPE on the CHSE cells similar to what has been described earlier (Karlsen et al., 2014). The temperature at which the bacterium was grown and the time of harvest (cell density) determined the severity of CPE. After growth at 6 °C, wild type supernatants harvested at OD_600_ of 6.0 showed highest CPE with complete lysis of the cells (Figs. 5A and 5B). Wild type supernatants harvested at OD_600_ of 6.0 after growth at 12 °C were less cytotoxic to the cells (Fig. 5A), but the CPE increased with increasing cell density, and after treatment with supernatants harvested at OD_600_ of 8.0 the cells suffered from severe CPE and few cells remained viable and attached to the substratum (Fig. 5C).

Compared to the negative control, some CPE was observed for cells treated with supernatants harvested from the Δ*litR* mutant, but most cells remained intact without losing the cell to cell contact (Fig. 5A). On the other hand, supernatants harvested at OD _600_ of 6.0 from the Δ*ainS* mutant grown at 6 °C induced severe CPE and few cells survived (Figs. 5A and 5B). However, after growth at 12 °C the supernatants harvested from the Δ*ainS* and Δ*litR* induced similar CPE and were less cytotoxic than the corresponding supernatants harvested from the wild type. Hence, in particular, QS and LitR play a role in regulation of CPE towards CHSE cells in *A. wodanis*, and this regulatory role is somewhat stronger at 6 °C compared to at 12 °C (Fig. 5, Table S3).

## Discussion

QS is known to regulate several phenotypes or traits in vibrios and aliivibrios such as motility, siderophore production, hemolysis, biofilm formation, protease production and virulence (Croxatto et al., 2002; Zhu et al., 2002; Sandy & Butler, 2009; Bjelland et al., 2012; Yang & Defoirdt, 2015; Elgaml & Miyoshi, 2017; Balado et al., 2018).

Before the study presented here, we had a limited knowledge regarding the QS systems in *A. wodanis.* However, from previous genome analysis and HPLC-MS/MS analyses of supernatants we knew that *A. wodanis* encoded two QS systems (LuxS/PQ and AinS/AinR) and produced one AHL (3OHC10-HSL) (Purohit et al., 2013; Hjerde et al., 2015). Thus, to explore the role of the QS in *A. wodanis*, the essential genes *ainS* and *litR*, were inactivated and their functional roles were investigated when the bacteria was grown at 6 °C and 12 °C.

Our study shows that AinS is the autoinducer synthase responsible for cell density dependent 3OHC10-HSL production in *A. wodanis*. This is similar to AinS in *A. salmonicida*, which produces the same type of AHL (3OHC10-HSL) (Hansen et al., 2015). Previous studies of pathogenic vibrios and aliivibrios have pointed to a relationship between a temperature closest to disease temperature and AHL production (Hansen et al., 2015; Bhedi et al., 2017). Similarly, the temperature was found to regulate 3OHC10-HSL production in *A. wodanis*, where the concentration was higher at 6 °C than at 12 °C. Thus, the effect of temperature on 3OHC10-HSL production in *A. wodanis* may correlate to the winter ulcer threshold temperature, 8 °C.

LitR in *A. salmonicida* and *A. fischeri* are activators of AHL production while the LitR homologue VanT in *Vibrio anguillarum* does not affect AHL production (Croxatto et al., 2002; Lupp & Ruby, 2004; Hansen et al., 2015). Interestingly, LitR is only a weak activator of 3OHC10-HSL synthesis in *A. wodanis*, suggesting that other mechanisms may be involved in regulation of AHL production. Several LitR-independent regulations such as AinS autoregulation or cyclic adenosine monophosphate (cAMP) - cAMP receptor protein (CRP)-, response regulator (GacA)-, posttranscriptional regulator (RsmA)- and regulator of general stress response (RpoS)- mediated regulation have been reported in *A. fischeri* (Lupp & Ruby, 2004; Lyell et al., 2013). The genome of *A. wodanis* encodes homologs of GacA (AWOD_I_1749), RpoS (AWOD_I_2147 and AWOD_II_1179), RsmA (AWOD_I_2393) and CRP (AWOD_I_0320) (Hjerde et al., 2015). However, further studies are needed to investigate if these regulators are involved in 3OHC10-HSL production in addition to LitR in *A.wodanis*.

The *A. wodanis* Δ*litR* mutant exhibited better growth than the wild type and Δ*ainS* mutant at the non-optimal temperatures 20 and 25 °C. At 20 °C the wild type and Δ*ainS* mutant stopped growing in the early log phase and then continued growing after few hours, whereas the Δ*litR* mutant grew well without this pause. This suggests that LitR is a negative regulator of growth and temporarily prevents growth of *A. wodanis* at 20 °C. Others have reported that QS upregulate growth at non-optimal temperatures such as in *A. fischeri* and *A. salmonicida* where deletion of *litR* led to slower growth than their respective wild types (Fidopiastis et al., 2002; Hansen et al., 2015). However, in some bacteria like *Pseudomonas aeruginosa*, the mutation of QS transcriptional regulators (LasR and RhlR) provided a growth advantage to the *lasR* and *rhlR* mutants over the wild type (Heurlier et al., 2005; Yan et al., 2007). Moreover, during alkaline stress, the *lasR* mutant in *P. aeruginosa* showed better cell viability than the wild type (Heurlier et al., 2005). Bacteria experience various fluctuations in the environment, and suboptimal temperature is a key stressor, which the bacteria have to react and respond to in order to survive. This is well-known from studies with *E.coli* where a temperature shift from 37 to 42 °C results in accumulation of heat shock proteins to maintain homeostasis and later, after the bacteria have adapted to the temperature, the heat shock proteins are down regulated to assist the growth again (Bukau, 1993; Guisbert et al., 2004). Thus, *A. wodanis* may respond to non-optimal temperatures by inducing heat shock proteins and start to grow again after adapting to the temperature shock. However, the mechanisms LitR may play in the response to stress and non-optimal temperatures needs to be further investigated.

*A. wodanis* is considered a secondary pathogen in winter ulcer disease, and little is known about virulence factors in this bacterium. In a community, bacteria produce various virulent and non-virulent factors that provide an opportunity for adaptation and survival, such as motility, biofilm formation, siderophore and protease production (Hibbing et al., 2010; Cullen & McClean, 2015; Diard & Hardt, 2017). In this study, we found that *A. wodanis* 06/09/139 was motile and produced siderophores, hemolysin, protease and chitinase. *A. wodanis* grows faster at 12 °C than at 6 °C, and the aforementioned phenotypes or activities were strongest at 12 °C. Deletion of *litR* and *ainS* in *A. wodanis* changed several phenotypes in this study. QS regulation of motility has been shown in numerous *Vibrionaceae* members, where the effect of QS on motility varies between bacteria. QS positively regulates motility in *V. harveyi* and *Vibrio cholerae*, whereas it negatively affects motility in *A. salmonicida, A. fischeri*, *Vibrio parahaemolyticus* and *Vibrio alginolyticus* (Lupp & Ruby, 2004; Nielsen et al., 2006; Rui et al., 2008; Bjelland et al., 2012; Kernell Burke et al., 2015; Yang & Defoirdt, 2015). Similarly, in our study, QS negatively regulates motility in *A. wodanis*. In a planktonic state, bacteria require higher motility to reach towards the host or surface, as they attach, the motility decreases to facilitate colonization (Lupp & Ruby, 2005; Liu et al., 2008). Since LitR negatively regulates motility, it is likely that *A. wodanis* is more motile at low cell density and reduces its motility as it reaches higher cell density by activating the LitR-AinS pathway. In *A. fischeri* and *V. cholerae*, hypermotility in QS mutants have led to low colonization of the hosts (Gardel & Mekalanos, 1996; Lupp et al., 2003; Lupp & Ruby, 2005). We speculate that the hypermotile Δ*ainS* and Δ*litR* strains may behave like planktonic cells and result in low colonization in the host.

The motility is often linked to biofilm formation and colony rugosity in many *Vibrio* and *Aliivibrio spp.* (Yildiz & Visick, 2009; Bjelland et al., 2012; Jemielita, Wingreen & Bassler, 2018; Khider et al., 2019). In the present study, neither the wild type nor the hypermotile strains (Δ*ainS* and Δ*litR*) formed biofilm or colony rugosity under the tested conditions.

Proteases play an essential role in numerous bacterial biological processes and also act as virulence factors in many pathogens (Rui et al., 2009; Syngkon et al., 2010). QS master regulators such as VanT in *V. anguillarum*, HapR in *V. cholera*, SmcR in *Vibrio vulnificus*, OpaR in *V. parahaemolyticus* are known to be associated with regulation of proteases (Croxatto et al., 2002; Wang et al., 2011; Elgaml & Miyoshi, 2017; Chang & Lee, 2018). However, in *A. wodanis* LitR did not affect protease production. Interestingly, AinS in *A. wodanis* seems to positively affect protease production. This observation suggests that protease production is activated by AinS independently of LitR. LitR independent regulations of proteases have also been reported in other bacteria (Chancey, Wood & Pierson, 1999; Elgaml & Miyoshi, 2017). As AHL is still produced in the Δ*litR* mutant, the AHL could bind to some unknown LitR-independent regulators and express the wild type proteases. Thus, there is a possible linkage between other regulators and the AHL 3OHC10-HSL.

Deletion of *litR* in *A. wodanis* led to a reduction in siderophores production and hemolytic activity. The bacterium secretes siderophores to acquire iron from the environment and is a potential virulent factor (Ratledge & Dover, 2000; Balado et al., 2018). The genome of *A. wodanis* encodes two siderophores clusters (AWOD_I_1553-1563 and AWOD_II_0923-0927) and several putative hemolysin genes (AWOD_I_0727, AWOD_I_2361, AWOD_I_2612, AWOD_II_0256 and AWOD_II_1158) with high similarity to *A. salmonicida* and *A. fischeri* MJ11 (Hjerde et al., 2015). Hemolysin and siderophores are under QS regulation in other *Vibrionaceae* members (Gao et al., 2018; McRose et al., 2018). The finding that LitR is a positive regulator of siderophore and hemolysin production suggests that these phenotypes are more significant at high cell densities in *A. wodanis*. However, consistent with the LitR regulation of AHL production, LitR seems only to be a weak activator of siderophore and hemolysin production and may include other regulation mechanisms. In *A. fischeri*, a mutation in *ainS* showed no effect on siderophore production (Lupp et al., 2003). Similarly, neither siderophore nor hemolysin production was affected in Δ*ainS* mutant, suggesting that their productions are not dependent on AHL-mediated QS system. Additionally, the performed hemolysis and proteases assays in this study are semi-quantified test and conducted mainly due to their importance in virulence. However, further experiments and quantification methods are required to draw a better conclusion. While QS is known to negatively regulate chitinase in *V. harveyi*, the QS does not affect the chitinase production in *A. fischeri* (Defoirdt et al., 2010; Cao et al., 2012). Like *A. fischeri*, deletion of *ainS* and *litR* in *A. wodanis* did not have an effect on chitinase production, suggesting the production is independent of the QS system.

In the study of Karlsen et al. supernatants (OD_600_ of 6.0–7.5) harvested from *A. wodanis* grown at 8  °C were cytotoxic to four different salmon cell lines including CHSE (Karlsen et al., 2014). Similarly, in the work presented here *A. wodanis* supernatants caused CPE on CHSE, but the severity varied with time of harvest and the temperature at which the bacterium was grown. When grown at 6 °C, a severe CPE was observed with supernatants harvested in the early in the stationary phase (OD_600_ of 6.0). However, when the cells were exposed to supernatants harvested at later stages in the stationary phase more cells survived and remained attached. This suggests that the factor(s) responsible for causing cell death is more strongly expressed early in the stationary phase at this temperature. The situation is opposite when the wild type was grown at 12 °C where a higher cell density seems to be vital for expression of the cytotoxic factor(s). Thus, if QS is involved in regulation of CPE the “quorum” needed to turn on this activity may be achieved at lower cell densities when the bacterium is grown at 6 °C compared to at 12  °C. Several pathogenic vibrios such as *V. cholerae*, *V. parahaemolyticus*, *V. vulnificus* and *V. alginolyticus* use QS to regulate cytotoxicity (Cao et al., 2010; Gode-Potratz & McCarter, 2011; Shao et al., 2011; Gao et al., 2018). Similarly, our results show that LitR and AinS are activators of cytopathogenicity. However, only the *litR* mutation led to reduced CPE when the CHSE cells were treated with supernatants harvested after growth at 6 °C suggesting that the cytotoxic effect is independent of AinS and AHL mediated QS at this temperature. In addition to the AinS/AinR system *A. wodanis* encodes the genes needed for the LuxS/LuxPQ system. Perhaps at 6 °C, the virulence or CPE is more dependent on this latter QS system or, so far, other unknown factors.

The temperature has been shown to regulate QS in some bacteria, where a difference in phenotypes between the wild type and QS mutants is clearly different at one temperature compared to another. This was seen for *A. salmonicida* where a *litR* mutation led to biofilm formation and rugose colonies when the bacteria were grown at a low temperature (Hansen et al., 2014). However, when the same bacteria were grown at higher temperatures the Δ*litR* mutant behaved like the wild type and was not able to produce biofilm and rugose colonies (Hansen et al., 2014). AinS in *A. salmonicida* produces the same AHL as *A. wodanis* in addition to seven LuxI produced AHLs, and the concentration of 3-OH-C10-HSL was much higher at low temperature (Hansen et al., 2015). *A. wodanis* is not able to produce rugose colonies or biofilm, and the difference in AHL production at different temperatures is modest. However, the different QS regulated phenotypes are expressed at 6 °C in *A. wodanis*, and at this temperature the CHSE cells showed highest CPE.

## Conclusion

Based on the findings presented in this study, *A. wodanis* 06/09/139 produces some virulent factors that may be used for inter- or intraspecies co-operation and competition for niche adaptations during winter ulcer development. Many bacteria use AHL-mediated QS for regulation of various phenotypic traits (Papenfort & Bassler, 2016). Like other *Vibrionaceae* members, the LuxS/PQ and AinS/AinR QS systems in *A. wodanis* probably convey into the same cascade to activate LitR and downstream genes. In this study, we found that AinS is responsible for autoinducer production. We have shown that temperature is an essential factor in regulating AHL production, growth and cytotoxicity. Although QS in *A. wodanis* may not be a crucial activator or repressor of virulence-associated phenotypic traits, the minor role in regulation can add knowledge to the winter ulcer disease development. The regulatory mechanisms other than QS that regulates the phenotypic traits in *A. wodanis* need to be further investigated.

##  Supplemental Information

10.7717/peerj.11980/supp-1 Supplemental Information 1 Schematic representation of *litR* and *ainS* gene context of *A. wodanis* 06/09/139 Pink blocks indicate the deleted region. Green arrows indicate the primer-binding sites. Black arrows indicate the transcription start sites. Green lines indicate the retained gene region. Gene position in the genome is presented in parentheses.

10.7717/peerj.11980/supp-2 Supplemental Information 2 Growth of wild type *A. wodanis* 06/09/139, Δ*ainS*, Δ*litR* and *litR*^+^ The strains were grown on BA 2.5 plates, at 25 °C for 2 days.

10.7717/peerj.11980/supp-3 Supplemental Information 3 Chitinase production of *A. wodanis* 06/09/139, Δ*ainS*, Δ*litR* and *litR*^+^ The bacterial cultures were spotted on colloidal chitin plates and incubated at 6 and 12 °C. The zones were measured after 4 days.

10.7717/peerj.11980/supp-4 Supplemental Information 4 Biofilm assay of wild type, Δ*ainS* and Δ*litR* in SWT media at 6 °C. Biofilm assay of wild type, Δ *ainS* and Δ *litR* in SWT media at 6 °. Cultures were visualized using Ziess Primo Vert microscope at 10x magnification and was photographed with AxioCam ERc5s after 2 days of incubation.

10.7717/peerj.11980/supp-5 Supplemental Information 5 Colony morphology of wild type, Δ*ainS* and Δ*litR* on SWT plates at 6 °C. Colony morphology of wild type, Δ*ainS* and Δ *litR* on SWT plates at 6 °C. (A) The colonies on 1.0%, 2.8% and 4.0% SWT plates were photographed after 3 days of incubation. (B) Colony morphology was visualized using Ziess Primo Vert microscope at 4x magnification and was photographed with AxioCam ERc5s after 2 weeks of incubation.

10.7717/peerj.11980/supp-6 Supplemental Information 6 Raw data for HPLC-MS/MS chromotograms, Fig. 1

10.7717/peerj.11980/supp-7 Supplemental Information 7 3OHC10-HSL concentrations produced by wild type *A. wodanis* 06/09/139, Δ*litR* and *litR*^+^ at different cell densities and temperatures

10.7717/peerj.11980/supp-8 Supplemental Information 8 Phenotypic activities tested for *A. wodanis* 06/09/139, Δ*ainS*, Δ*litR* and *litR*^+^ at 6 °C and 12 °C

10.7717/peerj.11980/supp-9 Supplemental Information 9 Absorbance measured after crystal violet staining of CHSE cells treated with supernatants harvested from strains grown at 6 °C and 12 °C

10.7717/peerj.11980/supp-10 Supplemental Information 10 Raw data for growth at 6 °C, 12 °C and 20 °C

10.7717/peerj.11980/supp-11 Supplemental Information 11 Raw data for 3OHC10-HSL concentrations produced by wild type A. wodanis 06/09/139, Δ*litR* and *litR*^+^ at different cell densities and temperatures

10.7717/peerj.11980/supp-12 Supplemental Information 12 Raw data for motility, siderophore, protease and hemolysis assays at 6 °C and 12 °C

10.7717/peerj.11980/supp-13 Supplemental Information 13 Raw data for absorbance measured after crystal violet staining of CHSE cells treated with supernatants harvested from strains grown at 6 °C and 12 °C

10.7717/peerj.11980/supp-14 Supplemental Information 14 Raw data for chitinase assay

10.7717/peerj.11980/supp-15 Supplemental Information 15 Sequencing of PCR products

## Abbreviations

AA: Peak area count
AHL: N-acyl-homoserine-lactone
3OHC10-HSL: N-3-hydroxy-decanoyl-homoserine-lactone
AI: Autoinducers
CAS: Chrome azurol S
cAMP: cyclic adenosine monophosphate
CHSE: Chinook Salmon Embryo Cells
CPE: Cytopathogenic Effect
CRP: cAMP receptor protein
HPLC-MS/MS: High performance liquid chromatography mass spectrometry
LB: Luria-Bertani
Mm: millimetre
OD_600_: Optical density measured at 600nm
PCR: Polymerase chain reaction
QS: Quorum Sensing
RT: Retention Time
WT: Wild type

## Additional Information and Declarations

### DNA Deposition

The following information was supplied regarding the deposition of DNA sequences: The sequences of mutations performed for genes litR AWOD_I_0419 and ainS AWOD_I_1040 are available in a Supplementary File. The *A.wodanis* 06/09/139 genome are available at NCBI: GCA_000953695.1, PRJEB6963, LN554848, LN554849, LN554847, LN554850, LN554851, LN554846.

### Data Availability

The following information was supplied regarding data availability: The raw measurements are available in the Supplemental Files.
