# Supplementary figures and images for: Quorum sensing in Aliivibrio wodanis 06/09/139 and its role in controlling various phenotypic traits
Source: PeerJ. 2021 Aug 24;9:e11980. doi: 10.7717/peerj.11980 (PMC8395575; doi:10.7717/peerj.11980)

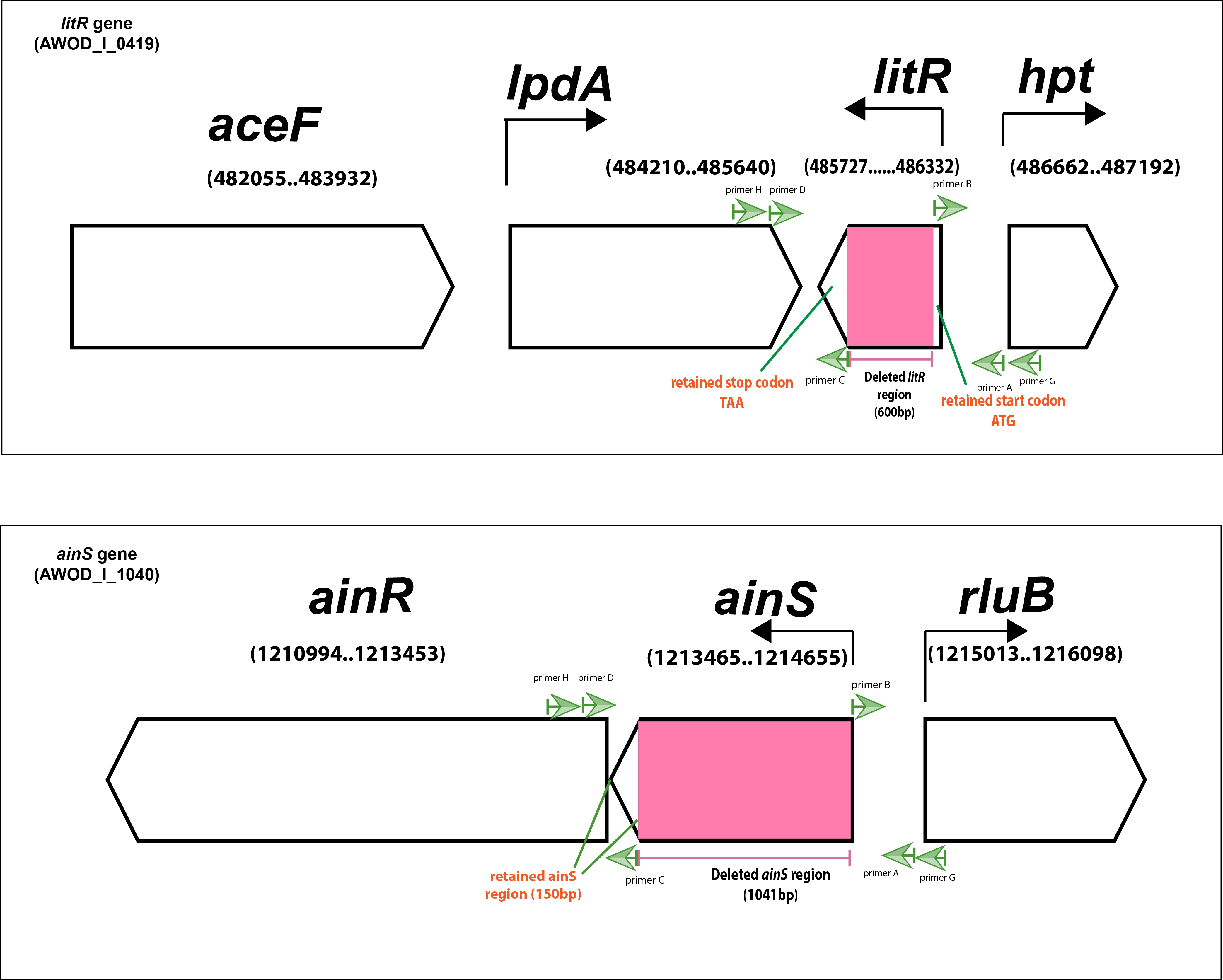

Supplement: Supplemental Information 1 — Pink blocks indicate the deleted region. Green arrows indicate the primer-binding sites. Black arrows indicate the transcription start sites. Green lines indicate the retained gene region. Gene position in the genome is presented in parentheses. [file peerj-09-11980-s001.png]

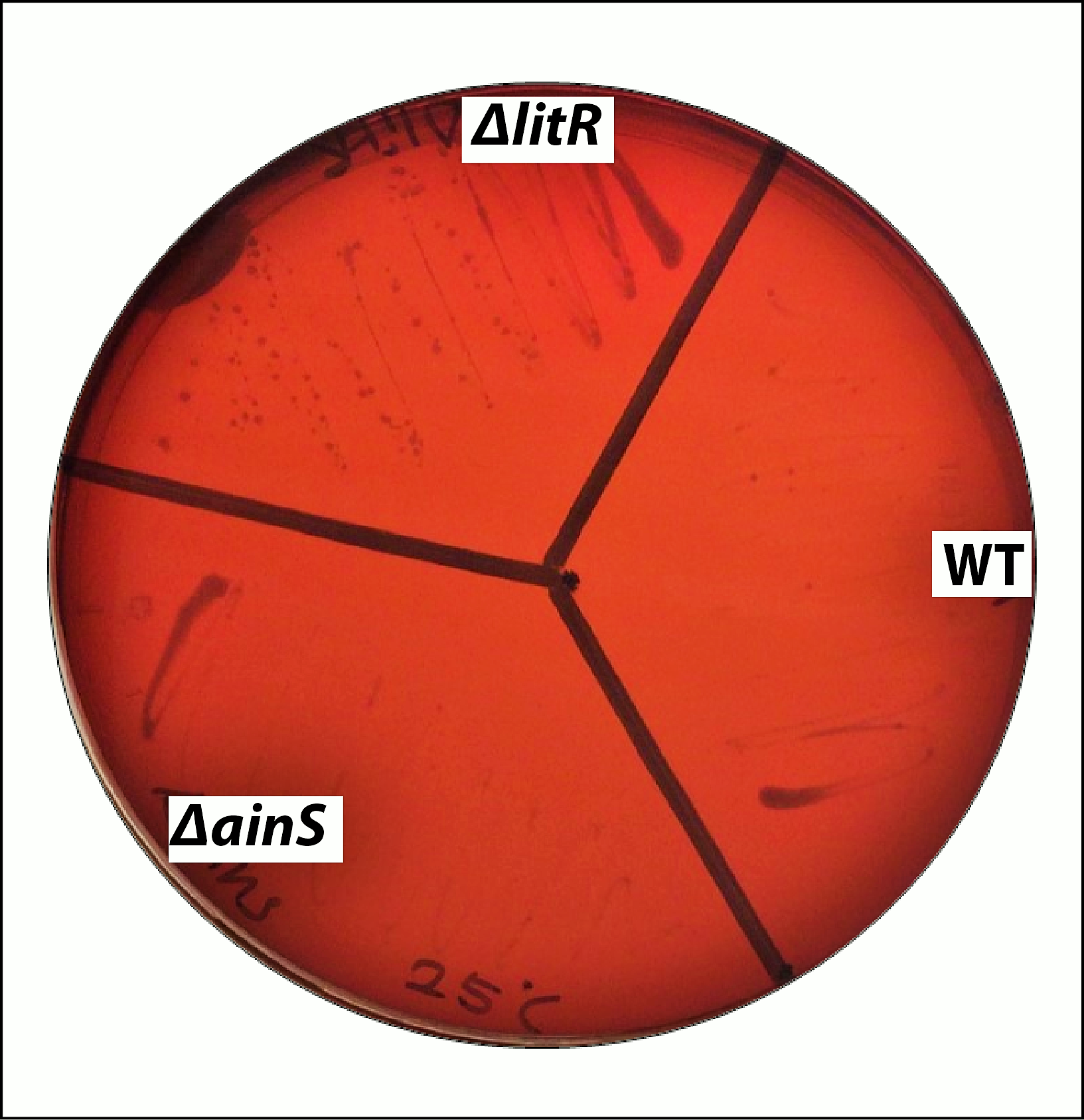

Supplement: Supplemental Information 2 [file peerj-09-11980-s002.png]

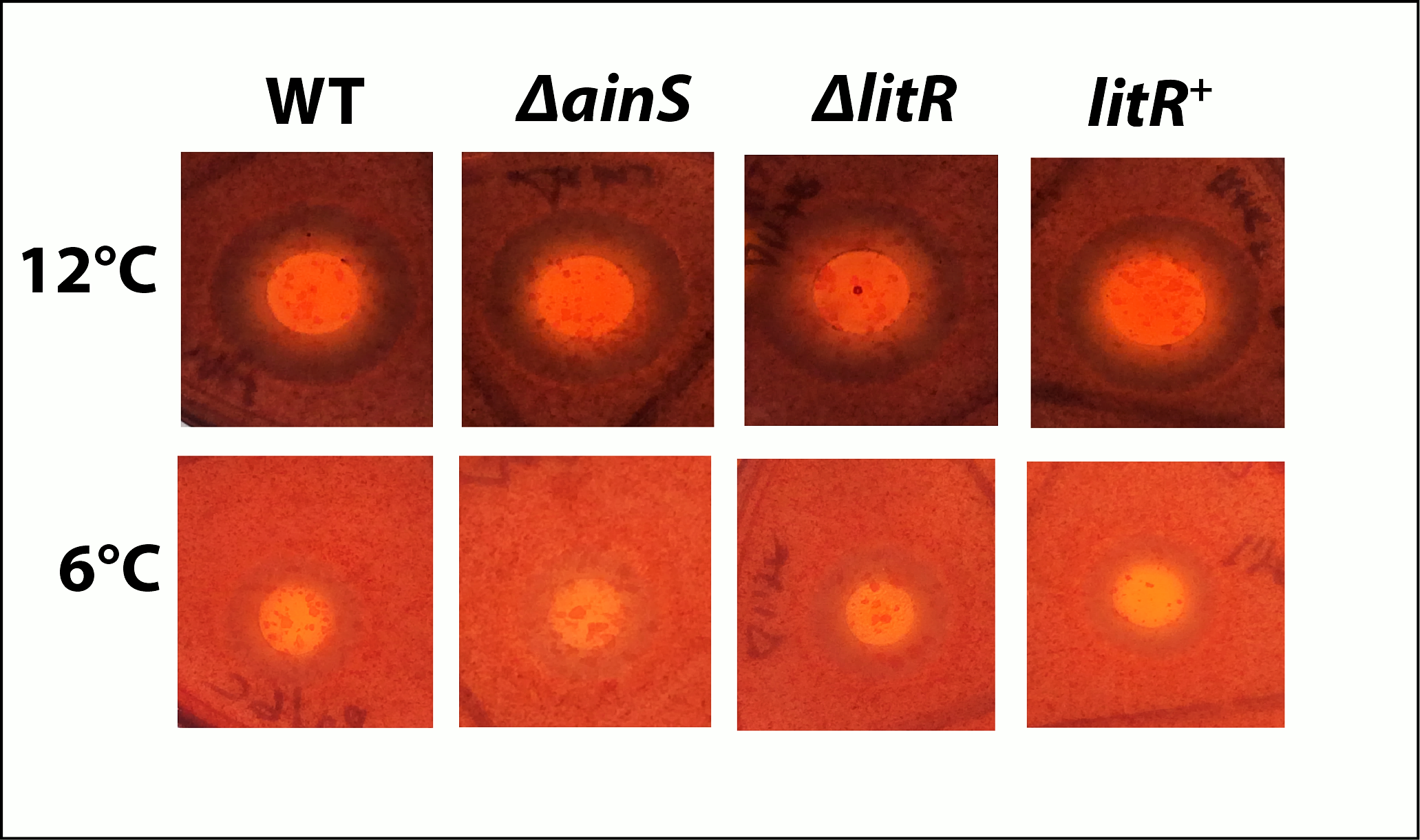

Supplement: Supplemental Information 3 [file peerj-09-11980-s003.png]

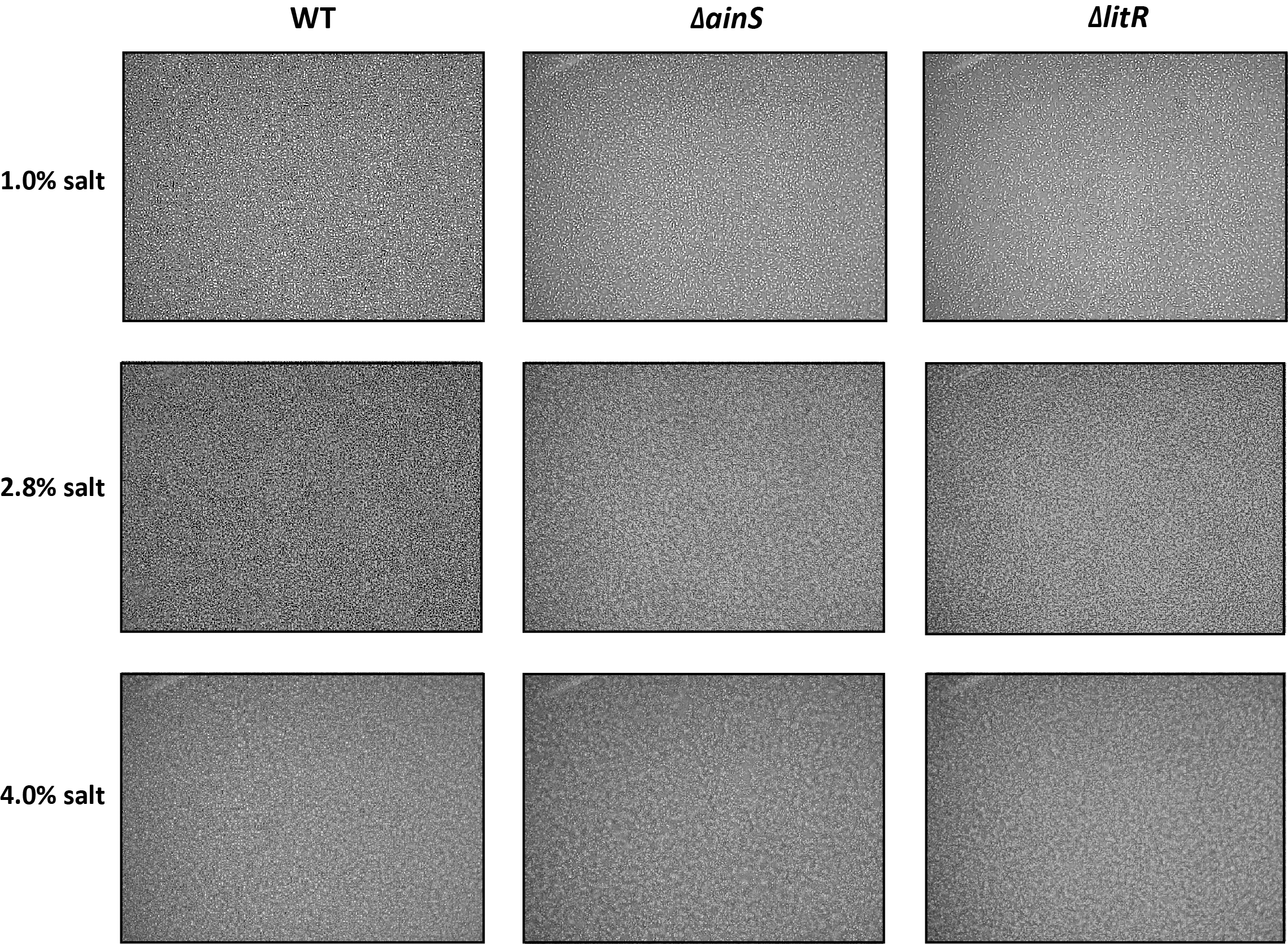

Supplement: Supplemental Information 4 — Biofilm assay of wild type, Δ ainS and Δ litR in SWT media at 6 °. Cultures were visualized using Ziess Primo Vert microscope at 10x magnification and was photographed with AxioCam ERc5s after 2 days of incubation. [file peerj-09-11980-s004.png]

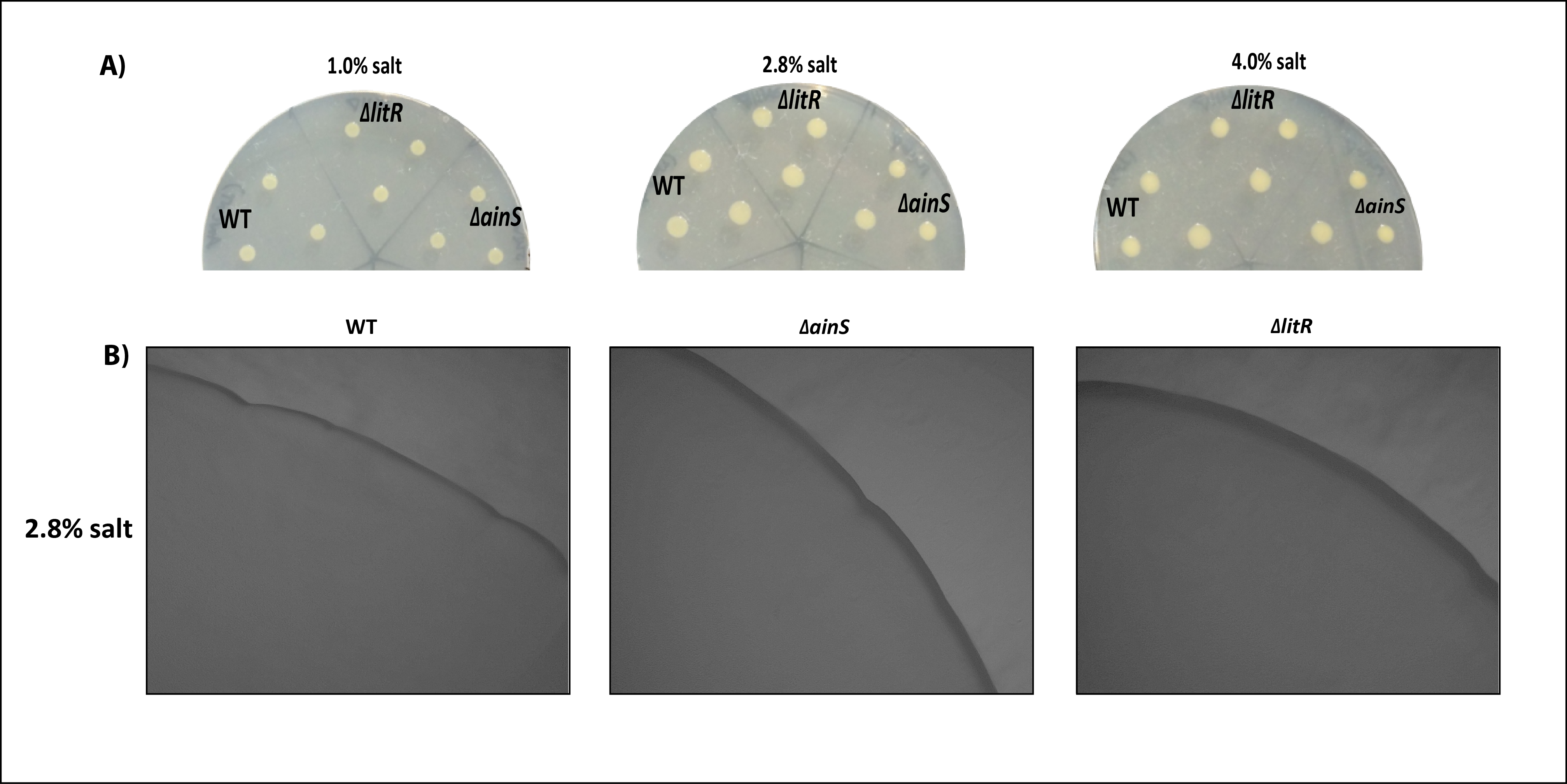

Supplement: Supplemental Information 5 — Colony morphology of wild type, ΔainS and Δ litR on SWT plates at 6 °C. (A) The colonies on 1.0%, 2.8% and 4.0% SWT plates were photographed after 3 days of incubation. (B) Colony morphology was visualized using Ziess Primo Vert microscope at 4x magnification and was photographed with AxioCam ERc5s after 2 weeks of incubation. [file peerj-09-11980-s005.png]
