# Supplementary material for: Quorum sensing in Aliivibrio wodanis 06/09/139 and its role in controlling various phenotypic traits
Source: PeerJ. 2021 Aug 24;9:e11980. doi: 10.7717/peerj.11980 (PMC8395575; doi:10.7717/peerj.11980)
Supplement: Supplemental Information 6 [file peerj-09-11980-s006.docx]

**Raw data Chromotograms for Fig.1**

**WT:**

**∆*ainS*:**

**∆*litR*:**

***litR^+^*:**
