# Supplementary material for: Quorum sensing in Aliivibrio wodanis 06/09/139 and its role in controlling various phenotypic traits
Source: PeerJ. 2021 Aug 24;9:e11980. doi: 10.7717/peerj.11980 (PMC8395575; doi:10.7717/peerj.11980)
Supplement: Supplemental Information 7 [file peerj-09-11980-s007.docx]

**Table S1. 3OHC10-HSL concentrations produced by wild type *A. wodanis* 06/09/139,** *Δ****litR* and *litR*^+^ at different cell densities and temperatures.**

|  | **3OHC10-HSL production (ng/ml/OD_600_)** | | | | | | |  |
| --- | --- | --- | --- | --- | --- | --- | --- | --- |
|  | **6°C** | | | **12°C** | | | |  |
| **OD_600nm_** | **WT** | ***ΔlitR*** | ***litR*^+^** | **WT** | | ***ΔlitR*** | ***litR^+^*** |  |
| 0.5 | 1.25± 0.14 | 1.05± 0.19 | 1.51± 0.11 | | 0.51± 0.10 | 0.37± 0.20 | 0.54± 0.23 | |
| 1.0 | 1.11± 0.14 | 0.92± 0.07 | 1.32± 0.16 | | 0.74± 0.09 | 0.50± 0.16 | 1.04± 0.18 | |
| 2.0 | 2.46± 0.53 | 1.61± 0.17 | 2.35± 0.24 | | 1.67± 0.11 | 1.37± 0.29 | 2.18± 0.14 | |
| 3.0 | 3.26± 1.88 | 2.25± 0.15 | 3.04± 0.16 | | 3.07± 0.39 | 2.01± 0.29 | 3.51± 0.16 | |
| 4.0 | 7.17± 0.49 | 4.21± 1.47 | 6.21± 0.46 | | 5.43± 0.52 | 3.14± 0.29 | 4.83± 0.43 | |
| 5.0 | 8.80± 0.24 | 7.17± 0.96 | 9.67± 0.05 | | 7.50± 1.89 | 6.27± 2.06 | 7.67± 0.50 | |
| 8.0 | 21.06± 0.43 | 16.01± 0.96 | 22.26± 0.17 | | 15.12± 0.94 | 11.78± 0.94 | 14.41± 0.91 | |
