# Supplementary material for: Quorum sensing in Aliivibrio wodanis 06/09/139 and its role in controlling various phenotypic traits
Source: PeerJ. 2021 Aug 24;9:e11980. doi: 10.7717/peerj.11980 (PMC8395575; doi:10.7717/peerj.11980)
Supplement: Supplemental Information 8 [file peerj-09-11980-s008.docx]

**Table S2. Phenotypic activities tested for *A. wodanis* 06/09/139, *ΔainS*,  *ΔlitR* and *litR^+^* at 6°C and 12°C.**

| **Phenotypes** | **WT** | | ***ΔainS*** | | ***ΔlitR*** | | ***litR^+^*** | |
| --- | --- | --- | --- | --- | --- | --- | --- | --- |
|  | **12°C** | **6°C** | **12°C** | **6°C** | **12°C** | **6°C** | **12°C** | **6°C** |
| Motility Zone  (mm) | 42.17  ± 3.19 | 18.00  ± 0.89 | 57.17  ± 3.87^†^ | 21.67  ±1.51^†^ | 57.67  ± 1.97^†^ | 24.58  ± 1.74^†^ | 42.67  ± 4.59 | 19.33  ± 0.52 |
|  |  |  |  |  |  |  |  |  |
| Siderophore Zone (mm) | 17.33  ± 1.15 | 14.00  ± 1.00 | 15.50  ± 1.32 | 13.67  ± 1.15 | 12.33  ± 1.15^†^ | 11.33  ± 1.53^†^ | 18.00  ± 2.00 | 13.67  ± 1.15 |
|  |  |  |  |  |  |  |  |  |
| Protease zone  ratio | 1.78± 0.15 | 1.47± 0.20 | 1.47± 0.07^†^ | 1.23± 0.08^†^ | 1.72± 0.10 | 1.42± 0.19 | 1.73± 0.06 | 1.42± 0.25 |
|  |  |  |  |  |  |  |  |  |
| Hemolytic zone  ratio | 1.84± 0.03 | 1.76± 0.07 | 1.81± 0.06 | 1.83± 0.07 | 1.66± 0.06^†^ | 1.58± 0.07^†^ | 1.74± 0.06 | 1.75± 0.04 |
|  |  |  |  |  |  |  |  |  |
| Chitinase zone  ratio | 2.28± 0.14 | 2.28± 0.12 | 2.23± 0.12 | 2.28± 0.12 | 2.28± 0.14 | 2.30± 0.10 | 2.28± 0.14 | 2.19± 0.20 |

^†^ Symbol denotes significant difference (*P* < 0.05) between *ΔlitR*/*ΔainS* mutants and wild type *A. wodanis*
