# Supplementary material for: Quorum sensing in Aliivibrio wodanis 06/09/139 and its role in controlling various phenotypic traits
Source: PeerJ. 2021 Aug 24;9:e11980. doi: 10.7717/peerj.11980 (PMC8395575; doi:10.7717/peerj.11980)
Supplement: Supplemental Information 9 [file peerj-09-11980-s009.docx]

**Table S3.** **Absorbance measured after crystal violet staining of CHSE cells treated with supernatants harvested from strains grown at 6°C and 12°C.**

|  | **Absorbance measured at 590nm** | | | | |
| --- | --- | --- | --- | --- | --- |
|  | **WT** | ***ΔainS*** | ***ΔlitR*** | ***litR^+^*** | **Negative control** |
| **12°C** |  |  |  |  |  |
| OD_600nm_ = 6.0 | 0.23 ± 0.04 | 0.27 ± 0.09 | 0.24 ± 0.04 | 0.19 ± 0.03 | 0.25 ± 0.02 |
| OD_600nm_ = 7.0 | 0.13 ± 0.02 | 0.20 ± 0.03 | 0.26 ± 0.03 | 0.12 ± 0.03 |  |
| OD_600nm_ = 8.0 | 0.10 ± 0.01 | 0.18 ± 0.04 | 0.19 ± 0.03 | 0.07 ± 0.01 |  |
|  |  |  |  |  |  |
| **6°C** |  |  |  |  |  |
| OD_600nm_ = 6.0 | 0.07 ± 0.01 | 0.09 ± 0.02 | 0.17 ± 0.01 | 0.07 ± 0.01 | 0.26 ± 0.02 |
| OD_600nm_ = 7.0 | 0.12 ± 0.02 | 0.15 ± 0.01 | 0.20 ± 0.02 | 0.10 ± 0.02 |  |
| OD_600nm_ = 8.0 | 0.17 ± 0.05 | 0.17 ± 0.03 | 0.20 ± 0.03 | 0.16 ± 0.04 |  |
