# Supplementary material for: Quorum sensing in Aliivibrio wodanis 06/09/139 and its role in controlling various phenotypic traits
Source: PeerJ. 2021 Aug 24;9:e11980. doi: 10.7717/peerj.11980 (PMC8395575; doi:10.7717/peerj.11980)
Supplement: Supplemental Information 15 [file peerj-09-11980-s015.pdf]

Alignment 1: *litR* and *ΔlitR* alignment
AWOD_I_0419 (*litR* Gene ID: 28539952 and flanking region sequence)
AM-01 (forward sequence)
AM-02 (complemented reverse sequence)

```
                   ....|....| ....|....| ....|....| ....|....| ....|....| ....|....|
                       5         15         25         35         45         55
AWOD_I_041   TACCACCTTA CCGATATAAG GTAGGTTACG ATATTTTTGT GCGTAGTCGA TACCAACACC
AM-01        ---------- ---------- ---------- ---------- ---------- ----------
AM-02        ---------- ---------- ---------- ---------- ---------- ----------

                   ....|....| ....|....| ....|....| ....|....| ....|....| ....|....|
                      65         75         85         95        105        115
AWOD_I_041   AACAACAAAC TCATCCGGAA TAGAAAAACC AATCCAATCC ACTTTAACAT CAACTTCACG
AM-01        ---------- ---------- ---------- ---------- ---------- ----------
AM-02        ---------- ---------- ---------- ---------- ---------- ----------

                   ....|....| ....|....| ....|....| ....|....| ....|....| ....|....|
                     125        135        145        155        165        175
AWOD_I_041   TCGAGAAGGT TTATCAAGCA GTGTACAAAT CGTAATTGAG TTTGGCTCAC GAATAGACAA
AM-01        ---------- ---------- ---------- ---------- ---------- ----------
AM-02        ---------- ---------- ---------- ---------- ---------- ----------

                   ....|....| ....|....| ....|....| ....|....| ....|....| ....|....|
                     185        195        205        215        225        235
AWOD_I_041   GATCTCACAC ACTTTACTTA GTGTATTACC GGTATCTATA ATGTCTTCTA CTAATAGGAT
AM-01        ---------- ---------- ---------- ---------- ---------- ----------
AM-02        ---------- ---------- ---------- ---------- ---------- ----------

                   ....|....| ....|....| ....|....| ....|....| ....|....| ....|....|
                     245        255        265        275        285        295
AWOD_I_041   GTCTTTACCT TTTATATCAT CATCAAGATC TTTCAAAATT CGTACGTCAC GCGTACTTTC
AM-01        ---------- ---------- ---------- ---------- ---------- ----------
AM-02        ---------- ---------- ---------- ---------- ---------- ----------

                   ....|....| ....|....| ....|....| ....|....| ....|....| ....|....|
                     305        315        325        335        345        355
AWOD_I_041   CATGCTATTT CCATAGCTTG ATGCTGTCAT AAAATCAACG GTCATTGGTG TATCAATCGC
AM-01        ---------- ---------- ---------- ---------- ---------- ----------
AM-02        ---------- ---------- ---------- ---------- ---------- ----------

                   ....|....| ....|....| ....|....| ....|....| ....|....| ....|....|
                     365        375        385        395        405        415
AWOD_I_041   TCGTGCTAAA TCAGCCATAA ATACAAATGA GCCACG-TAA TAAACCAATC ATCACTAAGC
AM-01        ---------- ---------- ---------- ---------- ---------- ---------A
AM-02        ---------- ---------- -------TGA GCCACGGTAA TAAACCAATC ATCACTAAGC

                   ....|....| ....|....| ....|....| ....|....| ....|....| ....|....|
                     425        435        445        455        465        475
AWOD_I_041   TATCAGACTC TTTATAATGT TCAGAAATCT GTTGACCTAA TTCTTTAACA CGTTGAGTAA
AM-01        CAT-AGACTC TTTATA-TGT TCAGAAATCT GTTGACCTAA TTCTTTAACA CGTTGAGTAA
AM-02        TATCAGACTC TTTATAATGT TCAGAAATCT GTTGACCTAA TTCTTTAACA CGTTGAGTAA

                   ....|....| ....|....| ....|....| ....|....| ....|....| ....|....|
                     485        495        505        515        525        535
AWOD_I_041   CCTCTTGTTC AGAGATCATC ACTTCTACTG TATGTTTCAT ACTGTCTCAC TTATTCGTTG
AM-01        CCTCTTGTTC AGAGATCATC ACTTCTACTG TATGTTTCAT ACTGTCTCAC TTATTCGTTG
AM-02        CCTCTTGTTC AGAGATCATC ACTTCTACTG TATGTTTCAT ACTGTCTCAC TTATTCGTTG

                   ....|....| ....|....| ....|....| ....|....| ....|....| ....|....|
                     545        555        565        575        585        595
AWOD_I_041   AGCCTACCTG CGGCAGGATT TAGATCTATA CAGTTTACAA CAAAAGCGCA CCTGAGATAG
AM-01        AGCCTACCTG CGGCAGGATT TAGATCTATA CAGTTTACAA CAAAAGCGCA CCTGAGATAG
AM-02        AGCCTACCTG CGGCAGGATT TAGATCTATA CAGTTTACAA CAAAAGCGCA CCTGAGATAG

                   ....|....| ....|....| ....|....| ....|....| ....|....| ....|....|
                     605        615        625        635        645        655
AWOD_I_041   GGACAATTAT AAAAGTTTTT CATACCTCTT TAGGATTAAA AGAAATTGTC TGATAAAATT
AM-01        GGACAATTAT AAAAGTTTTT CATACCTCTT TAGGATTAAA AGAAATTGTC TGATAAAATT
AM-02        GGACAATTAT AAAAGTTTTT CATACCTCTT TAGGATTAAA AGAAATTGTC TGATAAAATT
```

```
            ....|....| ....|....| ....|....| ....|....| ....|....| ....|....|
                  665        675        685        695        705        715
AWOD_I_041  AGTTAAAATA AAATGGTTTA TACGGTAAAC AACTGTAATT TTTTAAAATA ACCTTTAACC
AM-01       AGTTAAAATA AAATGGTTTA TACGGTAAAC AACTGTAATT TTTTAAAATA ACCTTTAACC
AM-02       AGTTAAAATA AAATGGTTTA TACGGTAAAC AACTGTAATT TTTTAAAATA ACCTTTAACC

            ....|....| ....|....| ....|....| ....|....| ....|....| ....|....|
                  725        735        745        755        765        775
AWOD_I_041  ACGTCATATT TTTAATTGAT AACATGAATA GTTGTTGAAT ATATAAACAG CCTTATGTAA
AM-01       ACGTCATATT TTTAATTGAT AACATGAATA GTTGTTGAAT ATATAAACAG CCTTATGTAA
AM-02       ACGTCATATT TTTAATTGAT AACATGAATA GTTGTTGAAT ATATAAACAG CCTTATGTAA

            ....|....| ....|....| ....|....| ....|....| ....|....| ....|....|
                  785        795        805        815        825        835
AWOD_I_041  GGTTACTGAG TAGAATAATA ATAAAACCCT ATATAAAATA ATAATTTGTT GGCAAGGATA
AM-01       GGTTACTGAG TAGAATAATA ATAAAACCCT ATATAAAATA ATAATTTGTT GGCAAGGATA
AM-02       GGTTACTGAG TAGAATAATA ATAAAACCCT ATATAAAATA ATAATTTGTT GGCAAGGATA

            ....|....| ....|....| ....|....| ....|....| ....|....| ....|....|
                  845        855        865        875        885        895
AWOD_I_041  TAAATAAATA TGGATACGAT CCAAAAAAGA CCGAGAACAA GGCTATCTCC AGAAAAGCGT
AM-01       TAAATAAATA TG--~~~~~~ ~~~~~~~~~~ ~~~~~~~~~~ ~~~~~~~~~~ ~~~~~~~~~~
AM-02       TAAATAAATA TG--~~~~~~ ~~~~~~~~~~ ~~~~~~~~~~ ~~~~~~~~~~ ~~~~~~~~~~

            ....|....| ....|....| ....|....| ....|....| ....|....| ....|....|
                  905        915        925        935        945        955
AWOD_I_041  AAAGAACAAC TTCTTGATAT TGCTATCGAG GTATTTTCAC AACGTGGTAT CGGCCGTGGC
AM-01       ~~~~~~~~~~ ~~~~~~~~~~ ~~~~~~~~~~ ~~~~~~~~~~ ~~~~~~~~~~ ~~~~~~~~~~
AM-02       ~~~~~~~~~~ ~~~~~~~~~~ ~~~~~~~~~~ ~~~~~~~~~~ ~~~~~~~~~~ ~~~~~~~~~~

            ....|....| ....|....| ....|....| ....|....| ....|....| ....|....|
                  965        975        985        995       1005       1015
AWOD_I_041  GGTCATGCTG ATGTTGCTGA AATAGCACAA GTATCAGTAG CAACTGTGTT TAACTATTTC
AM-01       ~~~~~~~~~~ ~~~~~~~~~~ ~~~~~~~~~~ ~~~~~~~~~~ ~~~~~~~~~~ ~~~~~~~~~~
AM-02       ~~~~~~~~~~ ~~~~~~~~~~ ~~~~~~~~~~ ~~~~~~~~~~ ~~~~~~~~~~ ~~~~~~~~~~

            ....|....| ....|....| ....|....| ....|....| ....|....| ....|....|
                 1025       1035       1045       1055       1065       1075
AWOD_I_041  CCTACAAGAG AAGATCTTGT TGATGACGTA TTAAACCGAG TAGAAGATGA GTTCCATTTA
AM-01       ~~~~~~~~~~ ~~~~~~~~~~ ~~~~~~~~~~ ~~~~~~~~~~ ~~~~~~~~~~ ~~~~~~~~~~
AM-02       ~~~~~~~~~~ ~~~~~~~~~~ ~~~~~~~~~~ ~~~~~~~~~~ ~~~~~~~~~~ ~~~~~~~~~~

            ....|....| ....|....| ....|....| ....|....| ....|....| ....|....|
                 1085       1095       1105       1115       1125       1135
AWOD_I_041  TTTGTTAAGA ATTCAATCTC TCTAGAGCTT GACGTTCGTA CAAATCTTCA CTCACTTTTA
AM-01       ~~~~~~~~~~ ~~~~~~~~~~ ~~~~~~~~~~ ~~~~~~~~~~ ~~~~~~~~~~ ~~~~~~~~~~
AM-02       ~~~~~~~~~~ ~~~~~~~~~~ ~~~~~~~~~~ ~~~~~~~~~~ ~~~~~~~~~~ ~~~~~~~~~~

            ....|....| ....|....| ....|....| ....|....| ....|....| ....|....|
                 1145       1155       1165       1175       1185       1195
AWOD_I_041  -ATGAATATT ATTGATGCTG TTCATTCAGA AAATAAATGG ATTAAAGTGT GGTTTGAATG
AM-01       ~~~~~~~~~~ ~~~~~~~~~~ ~~~~~~~~~~ ~~~~~~~~~~ ~~~~~~~~~~ ~~~~~~~~~~
AM-02       ~~~~~~~~~~ ~~~~~~~~~~ ~~~~~~~~~~ ~~~~~~~~~~ ~~~~~~~~~~ ~~~~~~~~~~

            ....|....| ....|....| ....|....| ....|....| ....|....| ....|....|
                 1205       1215       1225       1235       1245       1255
AWOD_I_041  GAGTACATCA ACGCGCGAAG AAGTTTGGCC TTTATTCTTA AGCAGCCATG TGAATAATCA
AM-01       ~~~~~~~~~~ ~~~~~~~~~~ ~~~~~~~~~~ ~~~~~~~~~~ ~~~~~~~~~~ ~~~~~~~~~~
AM-02       ~~~~~~~~~~ ~~~~~~~~~~ ~~~~~~~~~~ ~~~~~~~~~~ ~~~~~~~~~~ ~~~~~~~~~~

            ....|....| ....|....| ....|....| ....|....| ....|....| ....|....|
                 1265       1275       1285       1295       1305       1315
AWOD_I_041  AAAGATCATT CGCACAATGT TTGCTGAAGG TATAGAGCGA AATGAAGTGT GCAATGACCA
AM-01       ~~~~~~~~~~ ~~~~~~~~~~ ~~~~~~~~~~ ~~~~~~~~~~ ~~~~~~~~~~ ~~~~~~~~~~
AM-02       ~~~~~~~~~~ ~~~~~~~~~~ ~~~~~~~~~~ ~~~~~~~~~~ ~~~~~~~~~~ ~~~~~~~~~~

            ....|....| ....|....| ....|....| ....|....| ....|....| ....|....|
                 1325       1335       1345       1355       1365       1375
AWOD_I_041  TACTTCTGAA AATTTGGCAA AAATGCTACA CGGGATTTGT TATTCTGTAT TTATTCAAGC
AM-01       ~~~~~~~~~~ ~~~~~~~~~~ ~~~~~~~~~~ ~~~~~~~~~~ ~~~~~~~~~~ ~~~~~~~~~~
AM-02       ~~~~~~~~~~ ~~~~~~~~~~ ~~~~~~~~~~ ~~~~~~~~~~ ~~~~~~~~~~ ~~~~~~~~~~

            ....|....| ....|....| ....|....| ....|....| ....|....| ....|....|
                 1385       1395       1405       1415       1425       1435
AWOD_I_041  GAATCGTAGC TCTTCATCTG AAGAACTTGA AGAAACAGCT AATTGCTTCT TAAATATGTT
AM-01       ~~~~~~~~~~ ~~~~~~~~~~ ~~~~~~~~~~ ~~~~~~~~~~ ~~~~~~~~~~ ~~~~~~~~~~
```

```
AM-02         ~~~~~~~~~~ ~~~~~~~~~~ ~~~~~~~~~~ ~~~~~~~~~~ ~~~~~~~~~~ ~~~~~~~~~~

              ....|....| ....|....| ....|....| ....|....| ....|....| ....|....|
                 1445       1455       1465       1475       1485       1495
AWOD_I_041    ATGTATTTAT AAATAATATT CAGAAC~~~T CAGAAAGTAG ATAAT~~~~A AAAAACCGCT
AM-01         ~~~~~~~~~~ ~~~TAATATT CAGAAC---T CAGAAAGTAG ATAAT----A AAAAACCGCT
AM-02         ~~~~~~~~~~ ~~~TAATATT CAGAAC---T CAGAAAGTAG ATAAT----A AAAAACCGCT

              ....|....| ....|....| ....|....| ....|....| ....|....| ....|....|
                 1505       1515       1525       1535       1545       1555
AWOD_I_041    GA~~~CTAAT CAGCGGTTTT TTTATATTTA AATTCTAATG GTTTAAGAGA AATTATTTCT
AM-01         GA---CTAAT CAGCGGTTTT TTTATATTTA AATTCTAATG GTTTAAGAGA AATTATTTCT
AM-02         GA---CTAAT CAGCGGTTTT TTTATATTTA AATTCTAATG GTTTAAGAGA AATTATTTCT

              ....|....| ....|....| ....|....| ....|....| ....|....| ....|....|
                 1565       1575       1585       1595       1605       1615
AWOD_I_041    TTCTCTTTAC TGCTTTTGCA TTTGGAAGGT CAGTAATAGA ACCTTCATAA ATTTCAGCAG
AM-01         TTCTCTTTAC TGCTTTTGCA TTTGGAAGGT CAGTAATAGA ACCTTCATAA ATTTCAGCAG
AM-02         TTCTCTTTAC TGCTTTTGCA TTTGGAAGGT CAGTAATAGA ACCTTCATAA ATTTCAGCAG

              ....|....| ....|....| ....|....| ....|....| ....|....| ....|....|
                 1625       1635       1645       1655       1665       1675
AWOD_I_041    CCATACCAAT TGATTCATGC AATGTTGGGT GAGCATGGAT AGTTAGTGCG AT~~ATCTTC
AM-01         CCATACCAAT TGATTCATGC AATGTTGGGT GAGCATGGAT AGTTAGTGCG AT--ATCTTC
AM-02         CCATACCAAT TGATTCATGC AATGTTGGGT GAGCATGGAT AGTTAGTGCG AT--ATCTTC

              ....|....| ....|....| ....|....| ....|....| ....|....| ....|....|
                 1685       1695       1705       1715       1725       1735
AWOD_I_041    TGCATCACAA CCCATTTCGA TTGCAAGGCC AATTTCACCA AGAAGCTCAC CAGCATTAGT
AM-01         TGCATCACAA CCCATTTCGA TTGCAAGGCC AATTTCACCA AGAAGCTCAC CAGCATTAGT
AM-02         TGCATCACAA CCCATTTCGA T-GCAAGGCC AAT--CACCA AGAG---TAC CAGCTCC---

              ....|....| ....|....| ....|....| ....|....| ....|....| ....|....|
                 1745       1755       1765       1775       1785       1795
AWOD_I_041    ACCAACAATA GCACCACCGA TAACACGGTT AGTGTCTTTA TCAAAAAGAA GCTTAGTTAG
AM-01         ACCAACAATA GCACCACCGA ATAACACGA- ---------- ---------- ----------
AM-02         ---------- ---------- ---------- ---------- ---------- ----------

              ....|....| ....|....| ....|....| ....|....| ....|....| ....|....|
                 1805       1815       1825       1835       1845       1855
AWOD_I_041    ACCATCAGCA CAATCTGAAG CGATTGCACG GCCTGATGCA GCCCAAGGGA AGCTTGCTGC
AM-01         ---------- ---------- ---------- ---------- ---------- ----------
AM-02         ---------- ---------- ---------- ---------- ---------- ----------

              ....|....| ....|....| ....|....| ....|....| ....|....| ....|....|
                 1865       1875       1885       1895       1905       1915
AWOD_I_041    TTCGTAATTA ATACCTTCAG CTTTTGCTTC TTTTTCTGTT TTACCAACCC AAGCGACTTC
AM-01         ---------- ---------- ---------- ---------- ---------- ----------
AM-02         ---------- ---------- ---------- ---------- ---------- ----------

              ....|....| ....|....| ....|....| ....|....| ....|....| ....|....|
                 1925       1935       1945       1955       1965       1975
AWOD_I_041    TGGCTCAGTA TAAGCAATTG AAGGAATTAC TTTAGGATCG AAGTAGTGCT TCTTACCAGA
AM-01         ---------- ---------- ---------- ---------- ---------- ----------
AM-02         ---------- ---------- ---------- ---------- ---------- ----------

              ....|....| ....|....| ....|....| ....|....| ....|....| ....|....|
                 1985       1995       2005       2015       2025       2035
AWOD_I_041    AATAACTTCA GCAGCTACGT GACCTTCATG CACAC..... .......... ..........
AM-01         ---------- ---------- ---------- ---------- ---------- ----------
AM-02         ---------- ---------- ---------- ---------- ---------- ----------

              ....|....| ....|....| ....|....| ....|....| ....|....| ....|....|
                 2045       2055       2065       2075       2085       2095
AWOD_I_041    .......... .......... .......... .......... .......... ..........
AM-01         ---------- ---------- ---------- ---------- ---------- ----------
AM-02         ---------- ---------- ---------- ---------- ---------- ----------

              ....|....| ....|....| ....|....| ....|....| ....|....| ....|....|
                 2105       2115       2125       2135       2145       2155
AWOD_I_041    .......... .......... .......... .......... .......... ..........
AM-01         ---------- ---------- ---------- ---------- ---------- ----------
AM-02         ---------- ---------- ---------- ---------- ---------- ----------

              ....|....| ....|....| ....|....| ....|....| ....|....| ....|....|
                 2165       2175       2185       2195       2205       2215
AWOD_I_041    .......... .......... .......... .......... .......... ..........
```

```
AM-01         ---------- ---------- ---------- ---------- ---------- ----------
AM-02         ---------- ---------- ---------- ---------- ---------- ----------

              ....|....| ....|....| ....|....| ....|....| ....|....| ....|....|
                 2225       2235       2245       2255       2265       2275
AWOD_I_041    .......... .......... .......... .......... .......... ..........
AM-01         ---------- ---------- ---------- ---------- ---------- ----------
AM-02         ---------- ---------- ---------- ---------- ---------- ----------

              ....|....| ....|....| ....|....| ....|....| ....|....| ....|....|
                 2285       2295       2305       2315       2325       2335
AWOD_I_041    .......... .......... .......... .......... .......... ..........
AM-01         ---------- ---------- ---------- ---------- ---------- ----------
AM-02         ---------- ---------- ---------- ---------- ---------- ----------

              ....|....| ....|....| ....|....| ....|....| ....|....| ....|....|
                 2345       2355       2365       2375       2385       2395
AWOD_I_041    .......... .......... .......... .......... .......... ..........
AM-01         ---------- ---------- ---------- ---------- ---------- ----------
AM-02         ---------- ---------- ---------- ---------- ---------- ----------

              ....|....| ....|....| ....|....| ....|....| ....|....| ....|....|
                 2405       2415       2425       2435       2445       2455
AWOD_I_041    .......... .......... .......... .......... .......... ..........
AM-01         ---------- ---------- ---------- ---------- ---------- ----------
AM-02         ---------- ---------- ---------- ---------- ---------- ----------

              ....|....| ....|....| ....|....| ....|....| ....|....| ....|....|
                 2465       2475       2485       2495       2505       2515
AWOD_I_041    .......... .......... .......... .......... .......... ..........
AM-01         ---------- ---------- ---------- ---------- ---------- ----------
AM-02         ---------- ---------- ---------- ---------- ---------- ----------

              ....|....| ....|....| ....|....| ....|....| ....|....| ....|....|
                 2525       2535       2545       2555       2565       2575
AWOD_I_041    .......... .......... .......... .......... .......... ..........
AM-01         ---------- ---------- ---------- ---------- ---------- ----------
AM-02         ---------- ---------- ---------- ---------- ---------- ----------

              ....|....| ....|....| ..
                 2585       2595
AWOD_I_041    .......... .......... ..
AM-01         ---------- ---------- --
AM-02         ---------- ---------- --
```

Alignment 2: *litR* and *litR*$^+$ alignment
AWOD_I_0419 (*litR* and flanking region sequence)
AM-02 (*litR*$^+$ forward sequence)
AM-01 (*litR*$^+$ reverse complemented sequence replicate 1)
AM-3 (*litR*$^+$ reverse complemented sequence replicate 2)

```
              ....|....| ....|....| ....|....| ....|....| ....|....| ....|....|
                    5         15         25         35         45         55
AWOD_I_041    TACCACCTTA CCGATATAAG GTAGGTTACG ATATTTTTGT GCGTAGTCGA TACCAACACC
AM-02         ~~~~~~~~~~ ~~~~~~~~~~ ~~~~~~~~~~ ~~~~~~~~~~ ~~~~~~~~~~ ~~~~~~~~~~
AM-3          ---------- ---------- ---------- ---------- ---------- ----------
AM-01         ---------- ---------- ---------- ---------- ---------- ----------

              ....|....| ....|....| ....|....| ....|....| ....|....| ....|....|
                   65         75         85         95        105        115
AWOD_I_041    AACAACAAAC TCATCCGGAA TAGAAAAACC AATCCAATCC ACTTTAACAT CAACTTCACG
AM-02         ~~~~~~~~~~ ~~~~~~~~~~ ~~~~~~~~~~ ~~~~~~~~~~ ~~~~~~~~~~ ~~~~~~~~~~
AM-3          ---------- ---------- ---------- ---------- ---------- ----------
AM-01         ---------- ---------- ---------- ---------- ---------- ----------

              ....|....| ....|....| ....|....| ....|....| ....|....| ....|....|
                  125        135        145        155        165        175
AWOD_I_041    TCGAGAAGGT TTATCAAGCA GTGTACAAAT CGTAATTGAG TTTGGCTCAC GAATAGACAA
AM-02         ~~~~~~~~~~ ~~~~~~~~~~ ~~~~~~~~~~ ~~~~~~~~~~ ~~~~~~~~~~ ~~~~~~~~~~
AM-3          ---------- ---------- ---------- ---------- ---------- ----------
```

```
AM-01        ---------- ---------- ---------- ---------- ---------- ----------

             ....|....| ....|....| ....|....| ....|....| ....|....| ....|....|
                185        195        205        215        225        235
AWOD_I_041   GATCTCACAC ACTTTACTTA GTGTATTACC GGTATCTATA ATGTCTTCTA CTAATAGGAT
AM-02        ~~~~~~~~~~ ~~~~~~~~~~ ~~~~~~~~~~ ~~~~~~~~~~ ~~~~~~~~~~ ~~~~~~~~~~
AM-3         ---------- ---------- ---------- ---------- ---------- ----------
AM-01        ---------- ---------- ---------- ---------- ---------- ----------

             ....|....| ....|....| ....|....| ....|....| ....|....| ....|....|
                245        255        265        275        285        295
AWOD_I_041   GTCTTTACCT TTTATATCAT CATCAAGATC TTTCAAAATT CGTACGTCAC GCGTACTTTC
AM-02        ~~~~~~~~~~ ~~~~~~~~~~ ~~~~~~~~~~ ~~~~~~~~~~ ~~~~~~~~~~ ~~~~~~~~~~
AM-3         ---------- ---------- ---------- ---------- ---------- ----------
AM-01        ---------- ---------- ---------- ---------- ---------- ----------

             ....|....| ....|....| ....|....| ....|....| ....|....| ....|....|
                305        315        325        335        345        355
AWOD_I_041   CATGCTATTT CCATAGCTTG ATGCTGTCAT AAAATCAACG GTCATTGGTG TATCAATCGC
AM-02        ~~~~~~~~~~ ~~~~~~~~~~ ~~~~~~~~~~ ~~~~~~~~~~ ~~~~~~~~~~ ~~~~~~~~~~
AM-3         ---------- ---------- ---------- ---------- ---------- ----------
AM-01        ---------- ---------- ---------- ---------- ---------- ----------

             ....|....| ....|....| ....|....| ....|....| ....|....| ....|....|
                365        375        385        395        405        415
AWOD_I_041   TCGTGCTAAA TCAGCCATAA ATACAAATGA GCCACGTAAT AAACCAATCA TCACTAAGCT
AM-02        ~~~~~~~~~~ ~~~~~~~~~~ ~~~~~~~~~~ ~~~~~~~~~~ ~~~~~~~~~~ ~~~~~~~~~~
AM-3         ---------- ---------- ---------- ---------- ---------- ----------
AM-01        ---------- ---------- ---------- ---------- ---------- ---------C

             ....|....| ....|....| ....|....| ....|....| ....|....| ....|....|
                425        435        445        455        465        475
AWOD_I_041   ATCAGACTCT TTATAATGTT CAGAAATCTG TTGACCTAAT TCTTTAACAC GTTGAGTAAC
AM-02        ~~~~~~~~~~ ~~~~~~~~~~ ~~~~~~~~~~ ~~~~~~~~~~ ~~~~~~~~~~ ~~~~~~~~~~
AM-3         ---------- ---------- ---------- ---------- ---------- ----------
AM-01        AT-AGACTCT TTATA-TGTT CAGAAATCTG TTGACCTAAT TCTTTAACAC GTTGAGTAAC

             ....|....| ....|....| ....|....| ....|....| ....|....| ....|....|
                485        495        505        515        525        535
AWOD_I_041   CTCTTGTTCA GAGATCATCA CTTCTACTGT ATGTTTCATA CTGTCTCACT TATTCGTTGA
AM-02        ~~~~~~~~~~ ~~~~~~~~~~ ~~~~~~~~~~ ~~~~~~~~~~ ~~~~~~~~~~ ~~~~~~~~~~
AM-3         ---------- ---------- ---------- ---------- ---------- ----------
AM-01        CTCTTGTTCA GAGATCATCA CTTCTACTGT ATGTTTCATA CTGTCTCACT TATTCGTTGA

             ....|....| ....|....| ....|....| ....|....| ....|....| ....|....|
                545        555        565        575        585        595
AWOD_I_041   GCCTACCTGC GGCAGGATTT AGATCTATAC AGTTTACAAC AAAAGCGCAC CTGAGATAGG
AM-02        ~~~~~~~~~~ ~~~~~~~~~~ ~~~~~~~~CT CAGGATCTAT ACAGTTGCAA CAAAGCGCAC
AM-3         ---------- ---------- ---------- ---------- ---------- ----------
AM-01        GCCTACCTGC GGCAGGATTT AGATCTATAC AGTTTACAAC AAAAGCGCAC CTGAGATAGG

             ....|....| ....|....| ....|....| ....|....| ....|....| ....|....|
                605        615        625        635        645        655
AWOD_I_041   GACAATTATA AAAGTTTTTC ATACCTCTTT AGGATTAAAA GAAATTGTCT GATAAAATTA
AM-02        TGAGATAGGA CCATATAAAA GTTTTCATAC TCTTAGATAA AAGAATGTCT GATAAAATTA
AM-3         ---------- ---------- ---------- ---------- ---------- ----------
AM-01        GACAATTATA AAAGTTTTTC ATACCTCTTT AGGATTAAAA GAAATTGTCT GATAAAATTA

             ....|....| ....|....| ....|....| ....|....| ....|....| ....|....|
                665        675        685        695        705        715
AWOD_I_041   GTTAAAATAA AATGGTTTAT ACGGTAAACA ACTGTAATTT TTTAAAATAA CCTTTAACCA
AM-02        GTAAAATAAA ATGGTTATAC GGTAAACAAC TGTAATTTTT TAAAATAACC CTTTAAACCA
AM-3         ---------- ---------- ---------- ---------- ---------- ----------
AM-01        GTTAAAATAA AATGGTTTAT ACGGTAAACA ACTGTAATTT TTTAAAATAA CCTTTAACCA

             ....|....| ....|....| ....|....| ....|....| ....|....| ....|....|
                725        735        745        755        765        775
AWOD_I_041   CGTCATATTT TTAATTGATA ACATGAATAG TTGTTGAATA TATAAACAGC CTTATGTAAG
AM-02        CGTCATATTT TTAATTGATA ACATGAATAG TTGTTGAATA TATAAACAGC CTTATGTAAG
AM-3         ---------- ---------- ---------- ---------- ---------- ----------
AM-01        CGTCATATTT TTAATTGATA ACATGAATAG TTGTTGAATA TATAAACAGC CTTATGTAAG

             ....|....| ....|....| ....|....| ....|....| ....|....| ....|....|
                785        795        805        815        825        835
AWOD_I_041   GTTACTGAGT AGAATAATAA TAAAACCCTA TATAAAATAA TAATTTGTTG GCAAGGATAT
AM-02        GTTACTGAGT AGAATAATAA TAAAACCCTA TATAAAATAA TAATTTGTTG GCAAGGATAT
AM-3         ---------- ---------- ---------- ---------- ---------- ----------
```

```
AM-01        GTTACTGAGT AGAATAATAA TAAAACCCTA TATAAAATAA TAATTTGTTG GCAAGGATAT

             ....|....| ....|....| ....|....| ....|....| ....|....| ....|....|
                845        855        865        875        885        895
AWOD_I_041   AAATAAATAT GGATACGATC CAAAAAAGAC CGAGAACAAG GCTATCTCCA GAAAAGCGTA
AM-02        AAATAAATAT GGATACGATC CAAAAAAGAC CGAGAACAAG GCTATCTCCA GAAAAGCGTA
AM-3         ---------- ---------- ---------- ---------- ---------- ---GAGCGTA
AM-01        AAATAAATAT GGATACGATC CAAAAAAGAC CGAGAACAAG GCTATCTCCA GAAAAGCGTA

             ....|....| ....|....| ....|....| ....|....| ....|....| ....|....|
                905        915        925        935        945        955
AWOD_I_041   AAGAACAACT TCTTGATATT GCTATCGAGG TATTTTCACA ACGTGGTATC GGCCGTGGCG
AM-02        AAGAACAACT TCTTGATATT GCTATCGAGG TATTTTCACA ACGTGGTATC GGCCGTGGCG
AM-3         A~G~AC~ACT TCTTGATATT GCTATCGAGG TATTTTCACA ACGTGGTATC GGCCGTGGCG
AM-01        AAGAACAACT TCTTGATATT GCTATCGAGG TATTTTCACA ACGTGGTATC GGCCGTGGCG

             ....|....| ....|....| ....|....| ....|....| ....|....| ....|....|
                965        975        985        995        1005       1015
AWOD_I_041   GTCATGCTGA TGTTGCTGAA ATAGCACAAG T--ATCAGTA --GCAACTGT GTTTAACTAT
AM-02        GTCATGCTGA TGTTGCTGAA ATAGCACAAG T--ATCAGTA --GCAACTGT GTTTAACTAT
AM-3         GTCATGCTGA TGTTGCTGAA ATAGCACAAG T--ATCAGTA --GCAACTGT GTTTAACTAT
AM-01        GTCATGCTGA TGTTGCTGAA ATAGCACAAG T--ATCAGTA --GCAACTGT GTTTAACTAT

             ....|....| ....|....| ....|....| ....|....| ....|....| ....|....|
                1025       1035       1045       1055       1065       1075
AWOD_I_041   TTCCCTACAA GAGAAGATCT TGTTGATGAC GTATTAAACC GAGTAGAAGA TGAGTTCCAT
AM-02        TTCCCTACAA GAGAAGATCT TGTTGATGAC GTATTAAACC GAGTAGAAGA TGAGTTCCAT
AM-3         TTCCCTACAA GAGAAGATCT TGTTGATGAC GTATTAAACC GAGTAGAAGA TGAGTTCCAT
AM-01        TTCCCTACAA GAGAAGATCT TGTTGATGAC GTATTAAACC GAGTAGAAGA TGAGTTCCAT

             ....|....| ....|....| ....|....| ....|....| ....|....| ....|....|
                1085       1095       1105       1115       1125       1135
AWOD_I_041   TTATTTGTTA AGAATTCAAT CTCTCTAGAG C-TTGACGTT CGTACAAATC TTCACTCACT
AM-02        TTATTTGTTA AGAATTCAAT CTCTCTAGAG C~TTGACGTT CGTACAAATC TTCACTCACT
AM-3         TTATTTGTTA AGAATTCAAT CTCTCTAGAG C-TTGACGTT CGTACAAATC TTCACTCACT
AM-01        TTATTTGTTA AGAATTCAAT CTCTCTAGAG C-TTGACGTT CGTACAAATC TTCACTCACT

             ....|....| ....|....| ....|....| ....|....| ....|....| ....|....|
                1145       1155       1165       1175       1185       1195
AWOD_I_041   TTTAATGAAT ATTATTGATG CTGTTCATTC AGAAAATAAA TGGATTAAAG TGTGGTTTGA
AM-02        TTTAATGAAT ATTATTGATG CTGTTCATTC AGAAAATAAA TGGATTAAAG TGTGGTTTGA
AM-3         TTTAATGAAT ATTATTGATG CTGTTCATTC AGAAAATAAA TGGATTAAAG TGTGGTTTGA
AM-01        TTTAATGAAT ATTATTGATG CTGTTCATTC AGAAAATAAA TGGATTAAAG TGTGGTTTGA

             ....|....| ....|....| ....|....| ....|....| ....|....| ....|....|
                1205       1215       1225       1235       1245       1255
AWOD_I_041   ATGGAGTACA TCAACGCGCG AAGAAGTTTG GCCTTTATTC --TTAAGCAG CCATGTGAAT
AM-02        ATGGAGTACA TCAACGCGCG AAGAAGTTTG GCCTTTATTC ~~TTAAGCAG CCATGTGAAT
AM-3         ATGGAGTACA TCAACGCGCG AAGAAGTTTG GCCTTTATTC --TTAAGCAG CCATGTGAAT
AM-01        ATGGAGTACA TCAACGCGCG AAGAAGTTTG GCCTTTATTC --TTAAGCAG CCATGTGAAT

             ....|....| ....|....| ....|....| ....|....| ....|....| ....|....|
                1265       1275       1285       1295       1305       1315
AWOD_I_041   AATCAAAAGA TCATTCGCAC AATGTTTGCT GAAGGTATAG AGCGAAATGA AGTGTGCAAT
AM-02        AATCAAAAGA TCATTCGCAC AATGTTTGCT GAAGGTATAG AGCGAAATGA AGTGTGCAAT
AM-3         AATCAAAAGA TCATTCGCAC AATGTTTGCT GAAGGTATAG AGCGAAATGA AGTGTGCAAT
AM-01        AATCAAAAGA TCATTCGCAC AATGTTTGCT GAAGGTATAG AGCGAAATGA AGTGTGCAAT

             ....|....| ....|....| ....|....| ....|....| ....|....| ....|....|
                1325       1335       1345       1355       1365       1375
AWOD_I_041   GACCATACTT CTGAAAATTT GGCAAAAATG CTACACGGGA TTT----GTT ATTC-TGTAT
AM-02        GACCATACTT CTGAAAATTT GGCAAAAATG CTACACGGGA TTT----GTT ATTC-TGTAT
AM-3         GACCATACTT CTGAAAATTT GGCAAAAATG CTACACGGGA TTT----GTT ATTC-TGTAT
AM-01        GACCATACTT CTGAAAATTT GGCAAAAATG CTACACGGGA TTT----GTT ATTC-TGTAT

             ....|....| ....|....| ....|....| ....|....| ....|....| ....|....|
                1385       1395       1405       1415       1425       1435
AWOD_I_041   TTATTCAAGC GAATCGTAGC TCTTCATCTG AAGAACTTGA AGAAA--CAG CTAA-TTGCT
AM-02        TTATTCAAGC GAATCGTAGC TCTTCATCTG AAGAACTTGA AGAAA~~CAG CTAA~TTGCT
AM-3         TTATTCAAGC GAATCGTAGC TCTTCATCTG AAGAACTTGA AGAAA--CAG CTAA~TTGCT
AM-01        TTATTCAAGC GAATCGTAGC TCTTCATCTG AAGAACTTGA AGAAA--CAG CTAAATTGCT

             ....|....| ....|....| ....|....| ....|....| ....|....| ....|....|
                1445       1455       1465       1475       1485       1495
AWOD_I_041   TCTTAAATAT GTTATGTATT TA-TAAATAA TATTCAGAAC TCAGAAAGTA GATAATAAAA
AM-02        TCTTAAATAT GTTATGTATT TA~TAAATAA TATTCAGAAC TCAGAAAGTA GATAATAAAA
AM-3         TCTTAAATAT GTTATGTATT TA-TAAATAA TATTCAGAAC TCAGAAAGTA GATAATAAAA
```

```
AM-01        TCTTAA-TAT GTTATGTATT TA-TAA-TAA TATTCAGA-C TCAGAAAGTA GATA-TAAAA

             ....|....| ....|....| ....|....| ....|....| ....|....| ....|....|
                1505       1515       1525       1535       1545       1555
AWOD_I_041   AACCGCTGAC TAATCAGCGG TTTTTTTATA TTTAAATTCT AATGGTTTAA GAGAAATT-A
AM-02        AACCGCTGAC TAATCAGCGG TTTTTTTATA TTTAAATTCT AATGGTTTAA GAGAAATT~A
AM-3         AACCGCTGAC TAATCAGCGG TTTTTTTATA TTTAAATTCT AATGGTTTAA GAGAAATT-A
AM-01        AACGGCTGAA CTATCAGCGG TTTTTATATT AATCTATTGA GTCAGAGCAA TTGCATTCCA

             ....|....| ....|....| ....|....| ....|....| ....|....| ....|....|
                1565       1575       1585       1595       1605       1615
AWOD_I_041   TTTCTTTCTC --TTTACTGC TTTTGCATTT GGAAGGTCAG TAATAGAACC TTCATAAATT
AM-02        TTTCTTTCTC --TTTACTGC TTTTGCATTT GGAAGGTCAG TAATAGAACC TTCATAAATT
AM-3         TTTCTTTCTC --TTTACTGC TTTTGCATTT GGAAGGTCAG TAATAGAACC TTCATAAATT
AM-01        GTTCTCTTTA --CTGGCCAT TTTTGC---- ---------- ---------- ----------

             ....|....| ....|....| ....|....| ....|....| ....|....| ....|....|
                1625       1635       1645       1655       1665       1675
AWOD_I_041   TCAGCAGCCA TACCAATTGA TTCATGCAAT GTTGGGTGAG CATGGATAGT TAGTGCGATA
AM-02        TCAGCAGCCA TACCAATTGA TTCATGCAAT GTTGGGTGAG CATGGATAGT TAGTGCGATA
AM-3         TCAGCAGCCA TACCAATTGA TTCATGCAAT GTTGGGTGAG CATGGATAGT TAGTGCGATA
AM-01        ---------- ---------- ---------- ---------- ---------- ----------

             ....|....| ....|....| ....|....| ....|....| ....|....| ....|....|
                1685       1695       1705       1715       1725       1735
AWOD_I_041   TCTTCTGCAT CAC-AACCCA TTTCGATTGC AAGGCCAATT TCACCAAGAA GCTCACCAGC
AM-02        TCTTCTGCAT CAC-AACCCA TTTCGAT-GC AAGGCCAATC ACCAAGAGTC TCAGCTAC--
AM-3         TCTTCTGCAT CAC-AACCCA TTTCGATTGC AAGGCCAATT TCACCAAGAA GCTCACCAGC
AM-01        ---------- ---------- ---------- ---------- ---------- ----------

             ....|....| ....|....| ....|....| ....|....| ....|....| ....|....|
                1745       1755       1765       1775       1785       1795
AWOD_I_041   ATTAGTACCA ACAATAGCAC CACCGATAAC ACGGTTAGTG TCTTTATCAA AAAGAAGCTT
AM-02        ---------- ---------- ---------- ---------- ---------- ----------
AM-3         ATTAGTACCA ACAATAGCAC CACGCAAWWA CACGATTGGG RARSGKAAWA RWKTWAATGA
AM-01        ---------- ---------- ---------- ---------- ---------- ----------

             ....|....| ....|....| ....|....| ....|....| ....|....| ....|....|
                1805       1815       1825       1835       1845       1855
AWOD_I_041   AGTTAGACCA TCAGCACAAT CTGAAGCGAT TGCACGGCCT GAT-GCAGCC CAAGGGAAGC
AM-02        ---------- ---------- ---------- ---------- ---------- ----------
AM-3         SRTYGCGGGC GCRAAACWWT TTTTATAWAW WWTGGGGGAG WAT-RKWGWC AYSRGGGTCT
AM-01        ---------- ---------- ---------- ---------- ---------- ----------

             ....|....| ....|....| ....|....| ....|....| ....|....| ....|....|
                1865       1875       1885       1895       1905       1915
AWOD_I_041   TTGCTGCTTC GTAATTAAT- ----ACCTTC AGCTTTTGCT TCTTTTTCTG TTTTACCAAC
AM-02        ---------- ---------- ---------- ---------- ---------- ----------
AM-3         KSGTTMYCTG TAGATTGAGK GSSAGCTAAC TCSAWCCTYA CYTCCTGCGS GRTGMTWCWG
AM-01        ---------- ---------- ---------- ---------- ---------- ----------

             ....|....| ....|....| ....|....| ....|....| ....|....| ....|....|
                1925       1935       1945       1955       1965       1975
AWOD_I_041   CCAAGCGACT -TCTGGCTCA GTATAAGCAA TTGAAGGAAT TACTTTAGGA TCGAAGTAGT
AM-02        ---------- ---------- ---------- ---------- ---------- ----------
AM-3         ARAAAAAACG -ACKGASGTG CTYAGAGAGK WTGTTGWAGT ATTKATSATG TGYTCACRGM
AM-01        ---------- ---------- ---------- ---------- ---------- ----------

             ....|....| ....|....| ....|....| ....|....| ....|....| ....|....|
                1985       1995       2005       2015       2025       2035
AWOD_I_041   GCTTCTTACC AGAAATAACT TCAGCAGCTA CGTGACCTTC ATGCACAC-- ----------
AM-02        ---------- ---------- ---------- ---------- ---------- ----------
AM-3         ACAYCAGAGT KATGATAACA AMAGTCTGRC TGAMCGCYGG YSGTMTACTC AAGATGACAG
AM-01        ---------- ---------- ---------- ---------- ---------- ----------

             ....|....| ....|....| ....|....| ....|....| ....|....| ....|....|
                2045       2055       2065       2075       2085       2095
AWOD_I_041   ---------- ---------- ---------- ---------. .......... ..........
AM-02        ---------- ---------- ---------- ---------- ---------- ----------
AM-3         TTSRATGGTA TAAGAGATCG TCATCCTCCG KTSA-----. .......... ..........
AM-01        ---------- ---------- ---------- ---------. .......... ..........

             ....|....| ....|....| ....|....| ....|....| ....|....| ....|....|
                2105       2115       2125       2135       2145       2155
AWOD_I_041   .......... .......... .......... .......... .......... ..........
AM-02        ---------- ---------- ---------- ---------- ---------- ----------
AM-3         .......... .......... .......... .......... .......... ..........
```

```
AM-01          .......... .......... .......... .......... .......... ..........

               ....|....| ....|....| ....|....| ....|....| ....|....| ....|....|
                  2165       2175       2185       2195       2205       2215
AWOD_I_041     .......... .......... .......... .......... .......... ..........
AM-02          ---------- ---------- ---------- ---------- ---------- ----------
AM-3           .......... .......... .......... .......... .......... ..........
AM-01          .......... .......... .......... .......... .......... ..........

               ....|....| ....|....| ....|....| ....|....| ....|....| ....|....|
                  2225       2235       2245       2255       2265       2275
AWOD_I_041     .......... .......... .......... .......... .......... ..........
AM-02          ---------- ---------- ---------- ---------- ---------- ----------
AM-3           .......... .......... .......... .......... .......... ..........
AM-01          .......... .......... .......... .......... .......... ..........

               ....|....| ....|....| ....|....| ....|....| ....|....| ....|....|
                  2285       2295       2305       2315       2325       2335
AWOD_I_041     .......... .......... .......... .......... .......... ..........
AM-02          ---------- ---------- ---------- ---------- ---------- ----------
AM-3           .......... .......... .......... .......... .......... ..........
AM-01          .......... .......... .......... .......... .......... ..........

               ....|....| ....|....| ....|....| ....|....| ....|....| ....|....|
                  2345       2355       2365       2375       2385       2395
AWOD_I_041     .......... .......... .......... .......... .......... ..........
AM-02          ---------- ---------- ---------- ---------- ---------- ----------
AM-3           .......... .......... .......... .......... .......... ..........
AM-01          .......... .......... .......... .......... .......... ..........

               ....|....| ....|....| ....|....| ....|....| ....|....| ....|....|
                  2405       2415       2425       2435       2445       2455
AWOD_I_041     .......... .......... .......... .......... .......... ..........
AM-02          ---------- ---------- ---------- ---------- ---------- ----------
AM-3           .......... .......... .......... .......... .......... ..........
AM-01          .......... .......... .......... .......... .......... ..........

               ....|....| ....|....| ....|....| ....|....| ....|....| ....|....|
                  2465       2475       2485       2495       2505       2515
AWOD_I_041     .......... .......... .......... .......... .......... ..........
AM-02          ---------- ---------- ---------- ---------- ---------- ----------
AM-3           .......... .......... .......... .......... .......... ..........
AM-01          .......... .......... .......... .......... .......... ..........

               ....|....| ....|....| ....|....| ....|....| ....|....| ....|....|
                  2525       2535       2545       2555       2565       2575
AWOD_I_041     .......... .......... .......... .......... .......... ..........
AM-02          ---------- ---------- ---------- ---------- ---------- ----------
AM-3           .......... .......... .......... .......... .......... ..........
AM-01          .......... .......... .......... .......... .......... ..........

               ....|....| ....|....| ....|....| ....|....| ....|....|
                  2585       2595       2605       2615       2625
AWOD_I_041     .......... .......... .......... .......... ..........
AM-02          ---------- ---------- ---------- ---------- ----------
AM-3           .......... .......... .......... .......... ..........
AM-01          .......... .......... .......... .......... ..........
```

Alignment 3: *ainS* and *ΔainS* alignment

AWOD_I_1040 (*ainS* Gene ID: 28540605 and flanking region sequence)

AM-01 (*ΔainS* forward sequence)

AM-2 (*ΔainS* reverse complement sequence)

```
               ....|....| ....|....| ....|....| ....|....| ....|....| ....|....|
                     5         15         25         35         45         55
AWOD_I_104     CATCTAGACG ACCAACAGAT ACCCAACGAG AATCTTTGAT TTTTGGTAAA CGATCAAAAA
AM-01          ~~~~~~~~~~ ~~~~~~~~~~ ~~~~~~~~~~ ~~~~~~~~~~ ~~~~~~~~~~ ~~~~~~~~~~
```

```
AM-2         ~~~~~~~~~~ ~~~~~~~~~~ ~~~~~~~~~~ ~~~~~~~~~~ ~~~~~~~~~~ ~~~~~~~~~~

             ....|....| ....|....| ....|....| ....|....| ....|....| ....|....|
                  65         75         85         95        105        115
AWOD_I_104   CGGTACGACG ACCTTCAGGA TCGTGGCGAG TACATAACTC ACCTTCTGGT TTGTGGTAAG
AM-01        ~~~~~~~~~~ ~~~~~~~~~~ ~~~~~~~~~~ ~~~~~~~~~~ ~~~~~~~~~~ ~~~~~~~~~~
AM-2         ~~~~~~~~~~ ~~~~~~~~~~ ~~~~~~~~~~ ~~~~~~~~~~ ~~~~~~~~~~ ~~~~~~~~~~

             ....|....| ....|....| ....|....| ....|....| ....|....| ....|....|
                 125        135        145        155        165        175
AWOD_I_104   CAAGAACACG ACAGATTACT TCAGTAGACT CTTTTACCGA TACAACATGG CCATCGATAC
AM-01        ~~~~~~~~~~ ~~~~~~~~~~ ~~~~~~~~~~ ~~~~~~~~~~ ~~~~~~~~~~ ~~~~~~~~~~
AM-2         ~~~~~~~~~~ ~~~~~~~~~~ ~~~~~~~~~~ ~~~~~~~~~~ ~~~~~~~~~~ ~~~~~~~~~~

             ....|....| ....|....| ....|....| ....|....| ....|....| ....|....|
                 185        195        205        215        225        235
AWOD_I_104   GAATTTTCGC ATCAACATTT TCTAGGCGAT CACCTAGTTT TGCAATCTGG CCATCAACAC
AM-01        ~~~~~~~~~~ ~~~~~~~~~~ ~~~~~~~~~~ ~~~~~~~~~~ ~~~~~~~~~~ ~~~~~~~~~~
AM-2         ~~~~~~~~~~ ~~~~~~~~~~ ~~~~~~~~~~ ~~~~~~~~~~ ~~~~~~~~~~ ~~~~~~~~~~

             ....|....| ....|....| ....|....| ....|....| ....|....| ....|....|
                 245        255        265        275        285        295
AWOD_I_104   TAACACGATT AGACTGAATC ATCGTTTCTA ATTCACGACG AGAACCAAGA CCTGCTCGTG
AM-01        ~~~~~~~~~~ ~~~~~~~~~~ ~~~~~~~~~~ ~~~~~~~~~~ ~~~~~~~~~~ ~~~~~~~~~~
AM-2         ~~~~~~~~~~ ~~~~~TTTTT TTTTTTTTTY YYYMMSSRRR RRRRGCGAGA CCTGCTCGTG

             ....|....| ....|....| ....|....| ....|....| ....|....| ....|....|
                 305        315        325        335        345        355
AWOD_I_104   CTAATACTTT CTGTAATTTT TCGCTCATTT TTTACCTTAT TATGTCGCCT TCACAGGCGT
AM-01        ~~~~GYACTT TCTGTATTTT TCGCTCATTT TTTACCTTAT TATGTCGCCT TCACAGGCGT
AM-2         CTAATACTTT CTGTAATTTT TCGCTCATTT TTTACCTTAT TATGTCGCCT TCACAGGCGT

             ....|....| ....|....| ....|....| ....|....| ....|....| ....|....|
                 365        375        385        395        405        415
AWOD_I_104   CAATTAGCTT TTATTAATCA AGGACCTATC TGAATAAGAT AAGATTCCTC AATCACTTTA
AM-01        CAATTAGCTT TTATTAATCA AGGACCTATC TGAATAAGAT AAGATTCCTC AATCACTTTA
AM-2         CAATTAGCTT TTATTAATCA AGGACCTATC TGAATAAGAT AAGATTCCTC AATCACTTTA

             ....|....| ....|....| ....|....| ....|....| ....|....| ....|....|
                 425        435        445        455        465        475
AWOD_I_104   AAATTCTATA GAGGCTGATT ATACAATAAG GTTGTGACGA ATGGTAAAAA ATTAAGCACA
AM-01        AAATTCTATA GAGGCTGATT ATACAATAAG GTTGTGACGA ATGGTAAAAA ATTAAGCACA
AM-2         AAATTCTATA GAGGCTGATT ATACAATAAG GTTGTGACGA ATGGTAAAAA ATTAAGCACA

             ....|....| ....|....| ....|....| ....|....| ....|....| ....|....|
                 485        495        505        515        525        535
AWOD_I_104   AATTTATTTC ATGCTGACAT TTTCAGTATG ATGATAGAGC AGCTTATAAA ACAAGCTGCT
AM-01        AATTTATTTC ATGCTGACAT TTTCAGTATG ATGATAGAGC AGCTTATAAA ACAAGCTGCT
AM-2         AATTTATTTC ATGCTGACAT TTTCAGTATG ATGATAGAGC AGCTTATAAA ACAAGCTGCT

             ....|....| ....|....| ....|....| ....|....| ....|....| ....|....|
                 545        555        565        575        585        595
AWOD_I_104   TATATCTATC AAATAAATAA CTGAACAACA ATTGCACAAT TGAAATGCTC AAATATTTAT
AM-01        TATATCTATC AAATAAATAA CTGAACAACA ATTGCACAAT TGAAATGCTC AAATATTTAT
AM-2         TATATCTATC AAATAAATAA CTGAACAACA ATTGCACAAT TGAAATGCTC AAATATTTAT

             ....|....| ....|....| ....|....| ....|....| ....|....| ....|....|
                 605        615        625        635        645        655
AWOD_I_104   TTCAGGCTGA TATTACTTAA ACCAAAGGAG ATCCATATGA AATCGTTATA TAAAAAGTTA
AM-01        TTCAGGCTGA TATTACTTAA ACCAAAGGAG ATCCATATGA AATCGTTATA TAAAAAGTTA
AM-2         TTCAGGCTGA TATTACTTAA ACCAAAGGAG ATCCATATGA AATCGTTATA TAAAAAGTTA

             ....|....| ....|....| ....|....| ....|....| ....|....| ....|....|
                 665        675        685        695        705        715
AWOD_I_104   TCAACATTTG ATCTAAACAA TCTAGATGAA GGGGCACAAA AAAAATCAAC AAAATGAGTT
AM-01        TCAACATTTG ATCTAAACAA TCTAG~~~~~ ~~~~~~~~~~ ~~~~~~~~~~ ~~~~~~~~~~
AM-2         TCAACATTTG ATCTAAACAA TCTAG~~~~~ ~~~~~~~~~~ ~~~~~~~~~~ ~~~~~~~~~~

             ....|....| ....|....| ....|....| ....|....| ....|....| ....|....|
                 725        735        745        755        765        775
AWOD_I_104   TATAAAAAAG CTGTACGATT ACTGCATAAA ATCAAATACA CCGAACCTTT TACAACAATT
AM-01        ~~~~~~~~~~ ~~~~~~~~~~ ~~~~~~~~~~ ~~~~~~~~~~ ~~~~~~~~~~ ~~~~~~~~~~
AM-2         ~~~~~~~~~~ ~~~~~~~~~~ ~~~~~~~~~~ ~~~~~~~~~~ ~~~~~~~~~~ ~~~~~~~~~~

             ....|....| ....|....| ....|....| ....|....| ....|....| ....|....|
                 785        795        805        815        825        835
AWOD_I_104   AATTGAAGCT CGTAAACAGA CTATTTTATC TAAATTAAGT AACACAGATA CAGGTAACCT
```

```
AM-01        ~~~~~~~~~~ ~~~~~~~~~~ ~~~~~~~~~~ ~~~~~~~~~~ ~~~~~~~~~~ ~~~~~~~~~~
AM-2         ~~~~~~~~~~ ~~~~~~~~~~ ~~~~~~~~~~ ~~~~~~~~~~ ~~~~~~~~~~ ~~~~~~~~~~

             ....|....| ....|....| ....|....| ....|....| ....|....| ....|....|
                  845        855        865        875        885        895
AWOD_I_104   TTCGCATCTA TTTTCGACAG AAGAAGCCAT AGCTCTCATA GGGAAGCAAC CCTGCAATAT
AM-01        ~~~~~~~~~~ ~~~~~~~~~~ ~~~~~~~~~~ ~~~~~~~~~~ ~~~~~~~~~~ ~~~~~~~~~~
AM-2         ~~~~~~~~~~ ~~~~~~~~~~ ~~~~~~~~~~ ~~~~~~~~~~ ~~~~~~~~~~ ~~~~~~~~~~

             ....|....| ....|....| ....|....| ....|....| ....|....| ....|....|
                  905        915        925        935        945        955
AWOD_I_104   CTTACCTAAA CATTATTTTA TGATAGAGCA ACTTGCACAT AAATTATTTG GATGTTTATT
AM-01        ~~~~~~~~~~ ~~~~~~~~~~ ~~~~~~~~~~ ~~~~~~~~~~ ~~~~~~~~~~ ~~~~~~~~~~
AM-2         ~~~~~~~~~~ ~~~~~~~~~~ ~~~~~~~~~~ ~~~~~~~~~~ ~~~~~~~~~~ ~~~~~~~~~~

             ....|....| ....|....| ....|....| ....|....| ....|....| ....|....|
                  965        975        985        995       1005       1015
AWOD_I_104   AAGATGTTGG ACTGAGTTTG AAATTTTCCG AACCATTCAA CATTCATCAA ATAACTACCA
AM-01        ~~~~~~~~~~ ~~~~~~~~~~ ~~~~~~~~~~ ~~~~~~~~~~ ~~~~~~~~~~ ~~~~~~~~~~
AM-2         ~~~~~~~~~~ ~~~~~~~~~~ ~~~~~~~~~~ ~~~~~~~~~~ ~~~~~~~~~~ ~~~~~~~~~~

             ....|....| ....|....| ....|....| ....|....| ....|....| ....|....|
                 1025       1035       1045       1055       1065       1075
AWOD_I_104   CACATATTCT AAACAAGAGC ATATTAATTC GGAATCAAAC ATCATTGATG AAGATTATCA
AM-01        ~~~~~~~~~~ ~~~~~~~~~~ ~~~~~~~~~~ ~~~~~~~~~~ ~~~~~~~~~~ ~~~~~~~~~~
AM-2         ~~~~~~~~~~ ~~~~~~~~~~ ~~~~~~~~~~ ~~~~~~~~~~ ~~~~~~~~~~ ~~~~~~~~~~

             ....|....| ....|....| ....|....| ....|....| ....|....| ....|....|
                 1085       1095       1105       1115       1125       1135
AWOD_I_104   CTATGAAATA GTTAATGATA TTTCAAAGGA TAAACGTCTA TTTTATACCG ATTTTTGCGC
AM-01        ~~~~~~~~~~ ~~~~~~~~~~ ~~~~~~~~~~ ~~~~~~~~~~ ~~~~~~~~~~ ~~~~~~~~~~
AM-2         ~~~~~~~~~~ ~~~~~~~~~~ ~~~~~~~~~~ ~~~~~~~~~~ ~~~~~~~~~~ ~~~~~~~~~~

             ....|....| ....|....| ....|....| ....|....| ....|....| ....|....|
                 1145       1155       1165       1175       1185       1195
AWOD_I_104   ACAACCACTG CTACTATCCG ACGCCATTGT CTTAATAAAT ATAACAACAT TTATTAAAGA
AM-01        ~~~~~~~~~~ ~~~~~~~~~~ ~~~~~~~~~~ ~~~~~~~~~~ ~~~~~~~~~~ ~~~~~~~~~~
AM-2         ~~~~~~~~~~ ~~~~~~~~~~ ~~~~~~~~~~ ~~~~~~~~~~ ~~~~~~~~~~ ~~~~~~~~~~

             ....|....| ....|....| ....|....| ....|....| ....|....| ....|....|
                 1205       1215       1225       1235       1245       1255
AWOD_I_104   ACATAAATGG TATGAAATGC TTAGTATTCT TGACATCTCA TCAAAAGGAG AGCATTTTAT
AM-01        ~~~~~~~~~~ ~~~~~~~~~~ ~~~~~~~~~~ ~~~~~~~~~~ ~~~~~~~~~~ ~~~~~~~~~~
AM-2         ~~~~~~~~~~ ~~~~~~~~~~ ~~~~~~~~~~ ~~~~~~~~~~ ~~~~~~~~~~ ~~~~~~~~~~

             ....|....| ....|....| ....|....| ....|....| ....|....| ....|....|
                 1265       1275       1285       1295       1305       1315
AWOD_I_104   TCTGTACCAA TTAAAGACAA ACAATAGTTA CCCTACCATT ATTTCTTCAG CGCTTATTGA
AM-01        ~~~~~~~~~~ ~~~~~~~~~~ ~~~~~~~~~~ ~~~~~~~~~~ ~~~~~~~~~~ ~~~~~~~~~~
AM-2         ~~~~~~~~~~ ~~~~~~~~~~ ~~~~~~~~~~ ~~~~~~~~~~ ~~~~~~~~~~ ~~~~~~~~~~

             ....|....| ....|....| ....|....| ....|....| ....|....| ....|....|
                 1325       1335       1345       1355       1365       1375
AWOD_I_104   ACCATATCAA GACAAAGCAA -ACTGGCTGT TCTTTAATGA TTTCTTCCAA ACCAAACATT
AM-01        ~~~~~~~~~~ ~~~~~~~~~~ ~~~~~~~~~~ ~~~~~~~~~~ ~~~~~~~~~~ ~~~~~~~~~~
AM-2         ~~~~~~~~~~ ~~~~~~~~~~ ~~~~~~~~~~ ~~~~~~~~~~ ~~~~~~~~~~ ~~~~~~~~~~

             ....|....| ....|....| ....|....| ....|....| ....|....| ....|....|
                 1385       1395       1405       1415       1425       1435
AWOD_I_104   GGAAAAATGA AGGAGTAACA GATTCTTTAC TATCTTT--- TTTCCCTAAA CTGAAAACAA
AM-01        ~~~~~~~~~~ ~~~~~~~~~~ ~~~~~~~~~~ ~~~~~~~~~~ ~~~~~~~~~~ ~~~~~~~~~~
AM-2         ~~~~~~~~~~ ~~~~~~~~~~ ~~~~~~~~~~ ~~~~~~~~~~ ~~~~~~~~~~ ~~~~~~~~~~

             ....|....| ....|....| ....|....| ....|....| ....|....| ....|....|
                 1445       1455       1465       1475       1485       1495
AWOD_I_104   CATACTCTAA TGACCTCTCA -TCAGATAAT TCTAATTTAT CCAGTTCAGA TCTAGAAAAT
AM-01        ~~~~~~~~~~ ~~~~~~~~~~ ~~~~~~~~~~ ~~~~~~~~~~ ~~~~~~~~~~ ~~~~~~~~~~
AM-2         ~~~~~~~~~~ ~~~~~~~~~~ ~~~~~~~~~~ ~~~~~~~~~~ ~~~~~~~~~~ ~~~~~~~~~~

             ....|....| ....|....| ....|....| ....|....| ....|....| ....|....|
                 1505       1515       1525       1535       1545       1555
AWOD_I_104   GCTATTTTCT CTTCTATCAT AGACAAAAGA AGTGTGTGTG GAGTGATCCG ATTTACCATC
AM-01        ~~~~~~~~~~ ~~~~~~~~~~ ~~~~~~~~~~ ~~~~~~~~~~ ~~~~~~~~~~ ~~~~~~~~~~
AM-2         ~~~~~~~~~~ ~~~~~~~~~~ ~~~~~~~~~~ ~~~~~~~~~~ ~~~~~~~~~~ ~~~~~~~~~~

             ....|....| ....|....| ....|....| ....|....| ....|....| ....|....|
                 1565       1575       1585       1595       1605       1615
```

```
AWOD_I_104   AATGGACCAC GCCCTAAATT AAACCAAT-A TATGTATTTA GCTCAAAAAG GATTAGCCAA
AM-01        ~~~~~~~~~~ ~~~~~~~~~~ ~~~~~~~~~~ ~~~~~~~~~~ ~~~~~~~~~~ ~~~~~~~~~~
AM-2         ~~~~~~~~~~ ~~~~~~~~~~ ~~~~~~~~~~ ~~~~~~~~~~ ~~~~~~~~~~ ~~~~~~~~~~

             ....|....| ....|....| ....|....| ....|....| ....|....| ....|....|
                 1625       1635       1645       1655       1665       1675
AWOD_I_104   TGCACTCTAC AACTCAGGAA GAGATATGAT CTTTTCTATT ATAGAACAAC CCGCAATGGT
AM-01        ~~~~~~~~~~ ~~~~~~~~~~ ~~~~~~~~~~ ~~~~~~~~~~ ~~~~~~~~~~ ~~~~~~~~~~
AM-2         ~~~~~~~~~~ ~~~~~~~~~~ ~~~~~~~~~~ ~~~~~~~~~~ ~~~~~~~~~~ ~~~~~~~~~~

             ....|....| ....|....| ....|....| ....|....| ....|....| ....|....|
                 1685       1695       1705       1715       1725       1735
AWOD_I_104   TTTGTTTTAC CAAGCAATAA ATAATGATAA CTGCTATACA CCCTTTCTTT TTA--CGAGC
AM-01        ~~~~~~~~~~ ~~~~~~~~~~ ~~~~~~~~~~ ~~~~~~~~~~ ~~~~~~~~~~ ~~A~~CGAGC
AM-2         ~~~~~~~~~~ ~~~~~~~~~~ ~~~~~~~~~~ ~~~~~~~~~~ ~~~~~~~~~~ ~~A~~CGAGC

             ....|....| ....|....| ....|....| ....|....| ....|....| ....|....|
                 1745       1755       1765       1775       1785       1795
AWOD_I_104   CACCAAGATA TCAACAACTC AGGATTAATC ACATATAAAG GGATCTGCTT AACTAAGCAT
AM-01        CACCAAGATA TCAACAACTC AGGATTAATC ACATATAAAG GGATCTGCTT AACTAAGCAT
AM-2         CACCAAGATA TCAACAACTC AGGATTAATC ACATATAAAG GGATCTGCTT AACTAAGCAT

             ....|....| ....|....| ....|....| ....|....| ....|....| ....|....|
                 1805       1815       1825       1835       1845       1855
AWOD_I_104   ACAAGTAATG TGTTCAATCA ATATGATTTG AAATCTTATA ATGCTAAAAT TATAAAAAGA
AM-01        ACAAGTAATG TGTTCAATCA ATATGATTTG AAATCTTATA ATGCTAAAAT TATAAAAAGA
AM-2         ACAAGTAATG TGTTCAATCA ATATGATTTG AAATCTTATA ATGCTAAAAT TATAAAAAGA

             ....|....| ....|....| ....|....| ....|....| ....|....| ....|....|
                 1865       1875       1885       1895       1905       1915
AWOD_I_104   AGAAAAGATT TAAAACAACG ATGAGTTTCT TATAAATGTT AATTGGCTTC CCTAAAGATA
AM-01        AGAAAAGATT TAAAACAACG ATGAGTTTCT TATAAATGTT AATTGGCTTC CCTAAAGATA
AM-2         AGAAAAGATT TAAAACAACG ATGAGTTTCT TATAAATGTT AATTGGCTTC CCTAAAGATA

             ....|....| ....|....| ....|....| ....|....| ....|....| ....|....|
                 1925       1935       1945       1955       1965       1975
AWOD_I_104   ATCTATTGCT ATTTATTGCG GTATTCATTT TACTATGGGT AGCCTTCTTT ATCCGATCTT
AM-01        ATCTATTGCT ATTTATTGCG GTATTCATTT TACTATGGGT AGCCTTCTTT ATCCGATCTT
AM-2         ATCTATTGCT ATTTATTGCG GTATTCATTT TACTATGGGT AGCCTTCTTT ATCCGATCTT

             ....|....| ....|....| ....|....| ....|....| ....|....| ....|....|
                 1985       1995       2005       2015       2025       2035
AWOD_I_104   TATTTAAAGA TCGGATGGAG GTTGATCCTA AAATCTACTA CCCCTACATA GGGTATTCTA
AM-01        TATTTAAAGA TCGGATGGAG GTTGATCCTA AAATCTACTA CCCCTACATA GGGTATTCTA
AM-2         TATTTAAAGA TCGGATGGAG GTTGATCCTA AAATCTACTA CCCCTACATA GGGTATTCTA

             ....|....| ....|....| ....|....| ....|....| ....|....| ....|....|
                 2045       2055       2065       2075       2085       2095
AWOD_I_104   TTTTTATAGT CATTTGGATT TTAACCAACT TCTATTTTCA ATCTGAATTA TTATTAATCT
AM-01        TTTTTATAGT CATTTGGATT TTAACCAACT TCTATTTTCA ATCTGAATTA TTATTAATCT
AM-2         TTTTTATAGT CATTTGGATT TTAACCAACT TCTATTTTCA ATCTGAATTA TTATTAATCT

             ....|....| ....|....| ....|....| ....|....| ....|....| ....|....|
                 2105       2115       2125       2135       2145       2155
AWOD_I_104   TCAGTGAAAA AATTGCTCTT TATATGGCAA TAGCGGCCAA CCTCGCTTCA TATTTTGCTT
AM-01        TCAGTGAAAA AATTGCTCTT TATATGGCAA TAGCGGCCAA CCTCGCTTCA TATTTTGCTT
AM-2         TCAGTGAAAA AATTGCTCTT TATATGGCAA TAGCGGCCAA CCTCGCTTCA TATTTTGCTT

             ....|....| ....|....| ....|....| ....|....| ....|....| ....|....|
                 2165       2175       2185       2195       2205       2215
AWOD_I_104   TTGCTTTTGC CTTTCTTTTT TCTTGTCGGC TCGTATCTAC TAGAAAAGAG AAAAAAATGG
AM-01        TTGCTTTTGC CTTTCTTTTT TCTTGTCGGC TCGTATCTAC TAGAAAAGAG AAAAAAATGG
AM-2         TTGCTTTTGC CTTTCTTTTT TCTTGTCGGC TCGTATCTAC TAGAAAAGAG AAAAAAATGG

             ....|....| ....|....| ....|....| ....|....| ....|....| ....|....|
                 2225       2235       2245       2255       2265       2275
AWOD_I_104   CATCTTGGCA GGTAGTTTTA TTTTACGCAG AAACCGCCTT TGCTCTCGCT ATCAACCTAA
AM-01        CATCTTGGCA GGTAGTTTTA TTTTACGCAG AAACCGCCTT TGCTCTCGRT TYYYAYCTAA
AM-2         CATCTGGCAG GTAGTTATAT CAGC...... .......... .......... ..........

             ....|....| ....|....| ....|....| ....|....| ....|....| ....|....|
                 2285       2295       2305       2315       2325       2335
AWOD_I_104   TACCTAATTT AGCGGTTACT AATGTAACTA TTATTGATAA AGGTGTTTTT ACCATTAATT
AM-01        AAAA...... .......... .......... .......... .......... ..........
AM-2         .......... .......... .......... .......... .......... ..........

             ....|....| ....|....| ....|....| ....|....| ....|....| ....|....|
```

```
                2345       2355       2365       2375       2385       2395
AWOD_I_104   TTGGACCATT TGCTTCTATT TTCTTTATCA ATGCCTTTGT CTTTATTGGT TTAATCATCC
AM-01        .......... .......... .......... .......... .......... ..........
AM-2         .......... .......... .......... .......... .......... ..........

             ....|....| ....|....| ....|....| ....|....| ....|....| ....|....|
                2405       2415       2425       2435       2445       2455
AWOD_I_104   ATAATTTTTT ACAATTAAGA ACAAGTAAGC TAAAACTTAA CAAAGAAAAG TCCATTTACT
AM-01        .......... .......... .......... .......... .......... ..........
AM-2         .......... .......... .......... .......... .......... ..........

             ....|....| ....|....| ....|....| ....|....| ....|....| ....|....|
                2465       2475       2485       2495       2505       2515
AWOD_I_104   TATTATTTGG CATTCTTATT TTTATGTCTT CAACCATCAT ATCTCAAGTT ATTATTCCTA
AM-01        .......... .......... .......... .......... .......... ..........
AM-2         .......... .......... .......... .......... .......... ..........

             ....|....| ....|....| ....|....| ....|....| ....|....| ....|....|
                2525       2535       2545       2555       2565       2575
AWOD_I_104   TCGTATGGGG GGATTTCTCT TTTGCATGGC TTCCGCCCGC ACTTTCTGTA ACTGAATCCG
AM-01        .......... .......... .......... .......... .......... ..........
AM-2         .......... .......... .......... .......... .......... ..........

             ....|....| ....|....| ....|....| ....|....| ....|....| ....|....|
                2585       2595       2605       2615       2625       2635
AWOD_I_104   TCCTTATTGG TTATACCCTA CTCTTTCATA GATTATATAG CCTACGTTAT ATTTCATACT
AM-01        .......... .......... .......... .......... .......... ..........
AM-2         .......... .......... .......... .......... .......... ..........

             ....|....| ....|....| ....|....| ....|....| ....|....| ....|....|
                2645       2655       2665       2675       2685       2695
AWOD_I_104   GGGCATGCTC ATACACGATT AATATAACTA TATATCTAAT ACCTATTACT CTTATTTATT
AM-01        .......... .......... .......... .......... .......... ..........
AM-2         .......... .......... .......... .......... .......... ..........

             ....|....| ....|....| ....|....| ....|....| ....|....| ....|....|
                2705       2715       2725       2735       2745       2755
AWOD_I_104   TAACAAATGA CAATATCAAC TTACTATATA CTTGCATTTT TCTTATTATA TATACTGGGT
AM-01        .......... .......... .......... .......... .......... ..........
AM-2         .......... .......... .......... .......... .......... ..........

             ....|....| ....|....| ....|....| ....|....| ....|....| ....|....|
                2765       2775       2785       2795       2805       2815
AWOD_I_104   TGTTTTGGGA AAAAACACTT AAAAAATCAA AACTAGTTTC TAGTCTATTA ATTTACAAAA
AM-01        .......... .......... .......... .......... .......... ..........
AM-2         .......... .......... .......... .......... .......... ..........

             ....|....| ....|....| ....|....| ....|....| ....|....| ....|....|
                2825       2835       2845       2855       2865       2875
AWOD_I_104   ATAAGAAAAC ACCTGTAGAA GAAATATATG ATATTGCCGA TGAATTCAAA TACTCTAGTC
AM-01        .......... .......... .......... .......... .......... ..........
AM-2         .......... .......... .......... .......... .......... ..........

             ....|....| ....|....| ....|....| ....|....| ....|....| ....|....|
                2885       2895       2905       2915       2925       2935
AWOD_I_104   AGCATGCTAT GATCAAGTTA GCTACACTTC TAGATACTCC ACAAGAAGAA TTATTACTTG
AM-01        .......... .......... .......... .......... .......... ..........
AM-2         .......... .......... .......... .......... .......... ..........

             ....|....| ....|....| ....|....| ....|....| ....|....| ....|....|
                2945       2955       2965       2975       2985       2995
AWOD_I_104   TTGGAAAAAA TACTAATTAT AATATTTTTA TTCCACATCT TAATCAATCT AATTCGGCAC
AM-01        .......... .......... .......... .......... .......... ..........
AM-2         .......... .......... .......... .......... .......... ..........

             ....|....
                3005
AWOD_I_104   TAGTTAAAG
AM-01        .........
AM-2         .........
```
